# Modulation of astrocyte reactivity improves functional deficits in mouse models of Alzheimer’s disease

**DOI:** 10.1186/s40478-018-0606-1

**Published:** 2018-10-16

**Authors:** Kelly Ceyzériat, Lucile Ben Haim, Audrey Denizot, Dylan Pommier, Marco Matos, Océane Guillemaud, Marie-Ange Palomares, Laurene Abjean, Fanny Petit, Pauline Gipchtein, Marie-Claude Gaillard, Martine Guillermier, Sueva Bernier, Mylène Gaudin, Gwenaëlle Aurégan, Charlène Joséphine, Nathalie Déchamps, Julien Veran, Valentin Langlais, Karine Cambon, Alexis P Bemelmans, Jan Baijer, Gilles Bonvento, Marc Dhenain, Jean-François Deleuze, Stéphane H R Oliet, Emmanuel Brouillet, Philippe Hantraye, Maria-Angeles Carrillo-de Sauvage, Robert Olaso, Aude Panatier, Carole Escartin

**Affiliations:** 1 Commissariat à l’Energie Atomique et aux Energies Alternatives, Département de la Recherche Fondamentale, Institut de Biologie François Jacob, MIRCen, 92260 Fontenay-aux-Roses, France; 2 Centre National de la Recherche Scientifique, Université Paris-Sud, UMR 9199, Neurodegenerative Diseases Laboratory, 92260 Fontenay-aux-Roses, France; 3 Neurocentre Magendie, INSERM U1215, 33077 Bordeaux, France; 4 Université de Bordeaux, 33077 Bordeaux, France; 5 Commissariat à l’Energie Atomique et aux Energies Alternatives, Département de la Recherche Fondamentale, Institut de Biologie François Jacob, Centre National de Recherche en Génomique Humaine (CNRGH), F-91057 Evry, France; 6 7CEA-INSERM Université Paris-Diderot and Université Paris-Sud, Paris, France; 7 Université Paris-Diderot et Université Paris-Sud, Paris, France; 8 Present address: F.M. Kirby Neurobiology Center, Boston Children’s Hospital, and Department of Neurology, Harvard Medical School, Boston, USA

**Keywords:** Reactive astrocytes, Alzheimer’s disease, JAK2-STAT3 pathway, Signaling cascades, Viral vectors, Neuroinflammation, Mouse models

## Abstract

Astrocyte reactivity and neuroinflammation are hallmarks of CNS pathological conditions such as Alzheimer’s disease. However, the specific role of reactive astrocytes is still debated. This controversy may stem from the fact that most strategies used to modulate astrocyte reactivity and explore its contribution to disease outcomes have only limited specificity. Moreover, reactive astrocytes are now emerging as heterogeneous cells and all types of astrocyte reactivity may not be controlled efficiently by such strategies.

Here, we used cell type-specific approaches in vivo and identified the JAK2-STAT3 pathway, as necessary and sufficient for the induction and maintenance of astrocyte reactivity. Modulation of this cascade by viral gene transfer in mouse astrocytes efficiently controlled several morphological and molecular features of reactivity. Inhibition of this pathway in mouse models of Alzheimer’s disease improved three key pathological hallmarks by reducing amyloid deposition, improving spatial learning and restoring synaptic deficits.

In conclusion, the JAK2-STAT3 cascade operates as a master regulator of astrocyte reactivity in vivo. Its inhibition offers new therapeutic opportunities for Alzheimer’s disease.

**Electronic supplementary material:**

The online version of this article (10.1186/s40478-018-0606-1) contains supplementary material, which is available to authorized users.

## Introduction

Astrocytes become reactive in virtually all diseases of the central nervous system (CNS). Astrocyte reactivity is classically defined by two hallmarks: cellular hypertrophy and overexpression of intermediate filament proteins such as Glial Fibrillary Acidic Protein (GFAP) and vimentin [27]. But astrocyte reactivity involves significant transcriptional changes that go well beyond these two morphological alterations, and the functional consequences of reactivity are unclear [7, 9, 47, 67]. In Alzheimer’s disease (AD), the most frequent neurodegenerative disease (ND) characterized by amyloid and Tau deposition in the brain, as well as memory loss and synaptic alterations, the role of reactive astrocytes is still debated [13, 63].

Studies in cellular or mouse models of AD report changes in specific astrocyte functions, including metabolic support [2, 72], neurotransmitter recycling [51], antioxidant defense [2, 83] and synaptic transmission [31, 45]. However, the overall contribution of reactive astrocytes to AD remains unclear, because the changes reported in these studies can have beneficial, detrimental or mixed effects on neurons. Attempts to block astrocyte reactivity globally, through knock-out of astrocyte intermediate filament proteins [32, 38] or inhibition of intracellular signaling cascades [22, 77] show inconsistent effects on AD pathological outcomes. Such discrepancies could stem from the low efficiency and selectivity of these approaches that only impact astrocyte morphological changes or have partial effects on astrocyte reactivity. In addition, they may not target all reactive astrocyte populations. Indeed, reactive astrocytes display molecular and functional heterogeneity [3]. For example, two sub-types of reactive astrocytes termed A1 and A2 were recently described [48, 85]. Likewise, in AD, astrocytes in contact with amyloid plaques display stronger transcriptional changes than those distant to plaques [62]. To understand how reactive astrocytes contribute to AD, it is crucial to develop a new strategy that efficiently modulates all types of reactive astrocytes.

Several intracellular signaling cascades are traditionally associated with astrocyte reactivity in acute or chronic diseases, but it is not clear whether they directly control it in vivo see, [7, 33] for review. Recently, we showed that the transcription factor Signal Transducer and Activator of Transcription 3 (STAT3) is activated in reactive astrocytes of several murine and primate ND models [8]. It is also activated in acute CNS diseases and is involved in the formation of the glial scar [4, 26, 61]. STAT3 is the downstream effector of the Janus Kinase 2 (JAK2)-STAT3 pathway. In this cascade, the binding of several cytokines and growth factors to their receptors activate JAK2, leading to phosphorylation and nuclear accumulation of STAT3 [54]. STAT3 then activates the expression of multiple target genes, including GFAP in astrocytes [12, 54].

In this study, we aimed to 1) demonstrate the instrumental role of the JAK2-STAT3 pathway in controlling astrocyte reactivity in AD and 2) target this pathway to modulate astrocyte reactivity and evaluate its contribution to disease outcomes in AD mouse models. We establish that the JAK2-STAT3 pathway is a master regulator of astrocyte reactivity in vivo. It is necessary and sufficient for the induction and long-term maintenance of reactive astrocytes. Importantly, its inhibition blunts astrocyte reactivity, reduces amyloid deposition, improves spatial learning and restores synaptic function in AD mouse models, revealing that reactive astrocytes have mostly deleterious roles in AD.

## Methods

### Mice

Male APP/PS1dE9 transgenic mice (APP; http://jaxmice.jax.org/strain/005864.html) were injected at 3–4 month-old and studied 6 months later. Another cohort was injected at 15 month-old and studied 1 month later. APP mice harbor a chimeric mouse/human *App* gene with the Swedish mutations K595N and M596L (APPswe) and the human *Psen1* variant lacking exon 9 on a C57BL/6 J background [30]. Non-transgenic littermates were used as controls. Female triple transgenic (3xTg) mice express human APPswe and human Tau^P301L^ under a Thy-1 promoter as well as a point mutation on the mouse *Psen1* gene (PS1^M146V^), on a mixed C57BL/6 J x 129Sv background [59]. C57Bl/6 J x 129Sv mice were used as controls. They were injected at 3 month-old and studied at 8–9 month-old. Breeding pairs were obtained from the Mutant Mouse Regional Resource Centers. Finally, WT male C57Bl6/J mice were injected at 2 month-old and studied 1–4 months later. All experimental protocols were reviewed and approved by the local ethics committee (CETEA N°44) and submitted to the French Ministry of Education and Research (Approvals # APAFIS#4565–2016031711426915 v3, APAFIS#4503–2016031409023019). They were performed in a facility authorized by local authorities (authorization #B92–032-02), in strict accordance with recommendations of the European Union (2010–63/EEC). All efforts were made to minimize animal suffering and animal care was supervised by veterinarians and animal technicians skilled in rodent healthcare and housing. Animals were housed under standard environmental conditions (12-h light-dark cycle, temperature: 22 ± 1 °C and humidity: 50%) with *ad libitum* access to food and water. Mice of the appropriate genotype were randomly allocated to experimental groups.

### Viral vectors

We used adeno-associated virus (AAV2, serotype 9) that bear the gfaABC_1_D promoter, a synthetic promoter derived from the GFAP promoter [43], to drive specific transgene expression in astrocytes (Additional file 1: Figure S1). AAV were produced by our viral vector facility according to validated procedures [21]. Viral genome concentration in the vector batch was determined by qPCR on DNase resistant particles.

To modulate the JAK2-STAT3 pathway in mouse astrocytes, we generated AAV encoding murine SOCS3 or murine JAK2^T875N^, a constitutive active form of JAK2 (JAK2ca, [23]). Control viral vectors encoded GFP. AAV-SOCS3 or AAV-JAK2ca were co-injected with an AAV-GFP to visualize infected cells (same total viral titer). Depending on the experiment, bilateral injections of the same viruses were performed and controls were generated in different animals. Alternatively, the contralateral brain region was injected with the control virus and data analyzed with paired *t* test.

As a positive control for STAT1 immunoreactivity, we used mice injected with a lentiviral vector (LV) encoding the cytokine ciliary neurotrophic factor (CNTF), as previously described [18].

### Stereotactic injections

Mice were anesthetized with an *i.p.* injection of ketamine (100 mg/kg) and xylazine (10 mg/kg). For APP mice, xylazine was replaced by medetomidine (0.25 mg/kg) and anesthesia was reversed by an *s.c.* injection of atipamezole (0.25 mg/kg) at the end of the surgical procedure. Lidocaine (7 mg/kg) was injected subcutaneously at the incision site, 10 min prior to surgery. Mice received paracetamol in drinking water (1.6 mg/ml) for 48 h after surgery. Viral vectors were injected in the CA1 region of the hippocampus (coordinates from Bregma: anteroposterior (AP): − 2, lateral (L): +/− 2; ventral (V): − 1.2 mm from the dura; or more caudally as it improves viral diffusion AP: -3, L: +/− 3; V: -1.5 mm).

AAV were diluted in 0.1 M phosphate buffer saline (PBS) with 0.001% pluronic acid, at a final total concentration of 2.5 10^9^ viral genome (VG)/μl. LV were diluted in PBS with 1% bovine serum albumin (BSA), at a total final concentration of 100 ng p24/μl. Between 2 and 2.5 μl of viral suspensions were injected at a rate of 0.2 μl/min.

As a positive control for phospho-ERK immunoreactivity, 2 month-old C57BL/6 mice received unilateral intrastriatal injections of lipopolysaccharide (LPS) [1 μl at 5 mg/ml in PBS (*Escherichia coli*, serotype 055:B5; Sigma)] and analyzed seven days later.

### Immunohistology

**Fig. 1:**
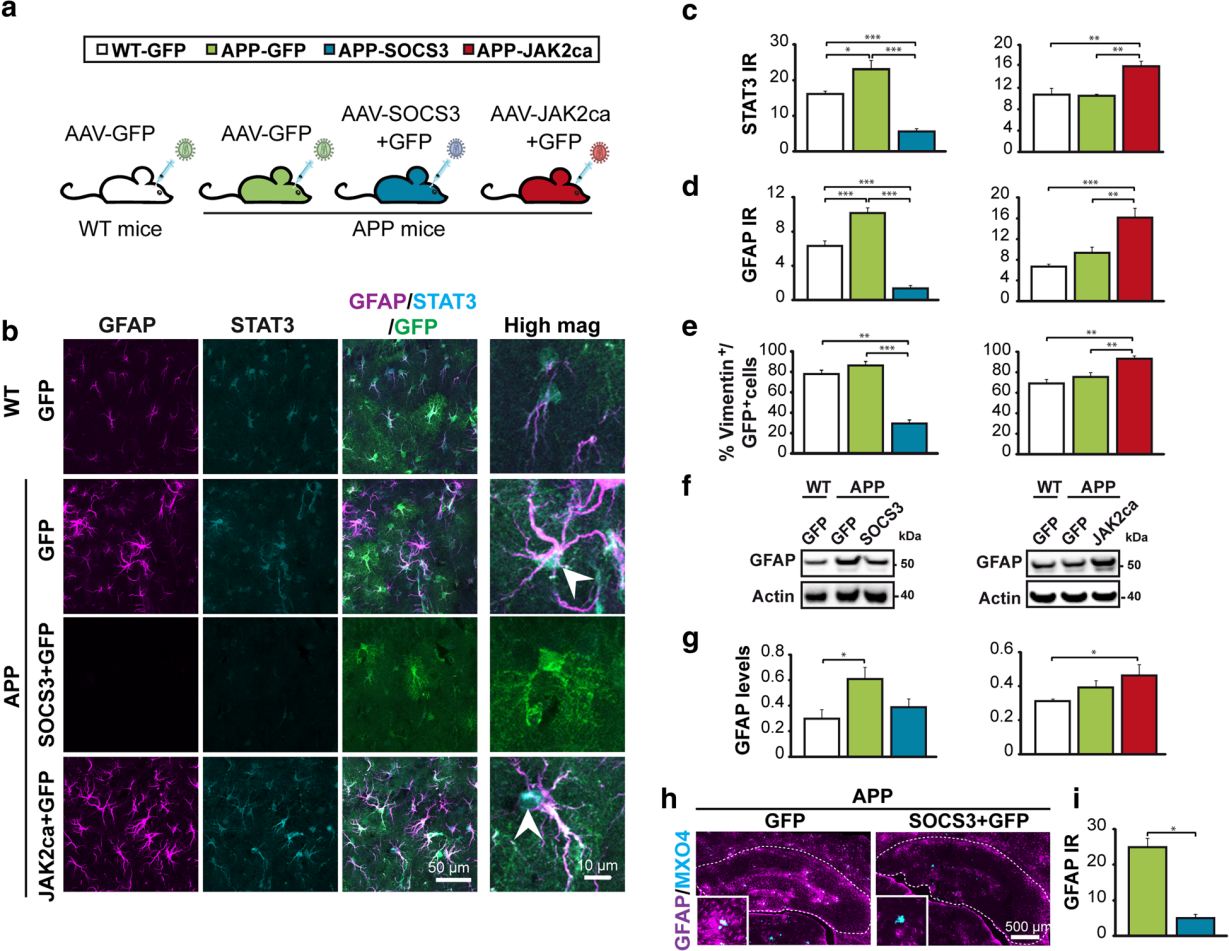
The JAK2-STAT3 pathway controls astrocyte reactivity in APP mice. **a**, Three month-old APP mice were injected in the hippocampus with AAV-GFP or AAV-SOCS3 + AAV-GFP. WT mice were injected with AAV-GFP. All mice were analyzed 6 months later. Another cohort was generated with AAV-JAK2ca used instead of AAV-SOCS3, to enhance astrocyte reactivity. **b**, Confocal images of astrocytes stained for GFAP (magenta) and STAT3 (cyan). In APP-GFP mice, astrocytes are hypertrophic, overexpress GFAP and show nuclear accumulation of STAT3 (indicating STAT3 activation, arrowhead), compared to WT-GFP mice. SOCS3 significantly reduces GFAP and STAT3 levels, whereas JAK2ca further increases GFAP and STAT3 levels in APP mice. **c, d**, Quantification of immunoreactivity (IR) for STAT3 (**c**, *N* = 4–5/group) and GFAP (**d**, *N* = 5–10/group) on immunostainings in **b**. **e**, The proportion of GFP^+^ astrocytes co-expressing vimentin is significantly lower in APP-SOCS3 mice and higher in APP-JAK2ca mice, than in control APP-GFP mice. *N* = 3–7/group. **f**, **g**, Western blot of GFAP in WT and APP mice shows the same modulation pattern of GFAP levels by SOCS3 and JAK2ca. GFAP levels were normalized by actin. Representative images and quantification from three independent membranes. *N* = 5–8/group. **h**, AAV-SOCS3 is also able to reverse astrocyte reactivity, when injected in the hippocampus of 15 month-old APP mice that already display severe plaque deposition (the hippocampus is outlined). **i**, GFAP IR quantification from images in **h**. *N* = 3/group. **c-e**, **g**, ANOVA and Tukey’s test. **i**, Paired *t* test. * *p* < 0.05, ** *p <* 0.01, *** *p <* 0.001

Some mice were killed by an overdose of sodium pentobarbital (180 mg/kg) and perfused with 4% paraformaldehyde (PFA) (Fig. 1h, 7). Alternatively, mice were killed by cervical dislocation and one brain hemisphere was rapidly dissected and drop-fixed in 4% PFA, while the other was used for biochemical analysis (Figs. 1b, 4, 6). In all cases, brains were post-fixed for 24 h in 4% PFA, cryoprotected in 30% sucrose solution and cut on a freezing microtome into 30 μm-thick coronal sections. Series of slices were stored at − 20 °C in an anti-freeze solution until used for immunostainings. All mice within a cohort were processed and analyzed in parallel with the exact same procedures.

*Immunofluorescence.* Slices were rinsed in PBS for 3 × 10 min and were blocked in 4.5% normal goat serum (NGS), 0.2% Triton X-100 in PBS (PBST) for 1 h at room temperature (RT). Slices were incubated overnight at 4 °C with the following primary antibodies diluted in 3% NGS/PBST: anti-GFAP-Cy3 (1:1,000; Sigma, #C9205), anti-GFAP (1:1,000, Rabbit, Dako, Troy, MI, #Z0334), anti-IBA1 (1:500, Rabbit, Wako, Richmond, VA, #019–19741), anti-MBP (1:500, Rabbit, Sigma, #M3821), anti-Olig2 (1:500, Rabbit, Millipore, #Ab9610), anti-S100β (1:500, Mouse, Sigma #S2532) or anti-Vimentin (1:1,000, Chicken, Abcam, Cambridge, UK, #ab24525).

Slices were rinsed 3 × 10 min in PBS and incubated with secondary Alexa Fluor-conjugated antibodies (Invitrogen, Carlsbad, CA) in 3% NGS/PBST for 1 h at RT. After 3 washes in PBS, slices were incubated overnight at 4 °C with an anti-GFP biotinylated antibody (1:500, Vector Laboratories, Burlingame, CA, #BA-0702) in 3% NGS/PBST. After 3 rinses with PBS, sections were incubated for 1 h at RT with Streptavidine-FITC (1:1,000, ThermoFisher Scientific, Waltham, MA, #SA100–02) in 3% NGS/PBST. Slices were rinsed three times with PBS before being mounted on SuperFrost® Plus (ThermoFisher Scientifc) slides and coverslipped with Fluorsave™ (Calbiochem, Darmstadt, Germany) or Fluormount™ (Sigma) medium.

For STAT3 and STAT1 stainings, slices were pretreated with 100% methanol for 10 min at − 20 °C and incubated with primary antibodies (STAT3α, 1:200, Rabbit, #8768P; STAT1, 1:400, Rabbit, #14994, Cell signaling, Danvers, MA) in SignalStain® antibody diluent (Cell signaling, #8112 L) for 72 h at 4 °C. For NeuN (1:500, Mouse, Chemicon, Billerica, MA, #MAB377) and BAM10 (1:1,000, Mouse, Sigma, #A3981) stainings, the mouse on mouse kit (MOM, Vector Laboratories) was used to reduce non-specific background, according to the manufacturer’s instructions. For Olig2 staining, a pre-treatment at 90 °C in Tris-EDTA Buffer pH = 9 (Diagnostic Biosystems, Pleasanton, CA) was performed. For *post-mortem* methoxy-XO4 (MXO4, Tocris, Bristol, UK) labeling, slices were incubated with 33 μg/ml MXO4 in 0.1 M PBS, for 30 min at RT under mild agitation. After 3 rinses with PBS, a standard protocol for immunofluorescence was performed as described above. Double or triple immunofluorescent stainings were performed successively, with each antibody incubated alone.

### Image analysis

GFAP immunoreactivity in the GFP^+^ area was quantified on 10×-tiled images of the hippocampus acquired with an epifluorescence microscope (Leica, Nussloch, Germany). Virally transduced GFP^+^ area was manually segmented and the corresponding GFAP mean signal was measured with Image J. Background signal was measured on unstained areas and subtracted to the GFAP total signal. The number and individual surface of BAM10 or MXO4-labelled plaques in the whole hippocampus were quantified on 10×-tiled fluorescent images with Image J. An automatic detection of objects with intensity and size thresholds was performed on serial sections, after manual segmentation of the hippocampus on each section.

Images were acquired on a Leica TCS SP8 confocal microscope. Stacked confocal images (10 to 18 z-steps of 1 μm, kept constant within a cohort, maximum intensity stack) were acquired on 3 slices per animal and 3 fields on each slice with a 40× objective. The number of vimentin^+^/GFP^+^ cells was manually counted in the GFP^+^ infected area, with Image J. STAT3^+^ cell bodies were manually segmented and the mean grey value for STAT3 IR was measured in each cell.

A Sholl analysis was performed on GFAP^+^ hippocampal astrocytes to quantify morphological parameters, with an Image J plugin [20] and defined radius parameters (starting: 5 μm; ending: 100 μm; step: 5 μm). GFAP immunofluorescent staining was detected with Image J threshold function on maximum projection confocal z-stack images (40×, average 12 steps, 1 mm step, zoom 1). This analysis requires detectable GFAP levels and therefore could not be conducted on APP-SOCS3 astrocytes that display very low GFAP expression.

For quantification of MXO4-labelled Aβ in microglia, stacks of 0.4 μm confocal images covering the entire height of the plaque were acquired on 15 MXO4^+^ plaques per animal (3–4 plaques/slice). The number of IBA1^+^ microglial cells in direct contact with MXO4^+^ plaques was counted manually in the acquired volume and the localization of MXO4^+^ material (membrane or soma) was determined.

### Protein extraction

Mice were killed by an overdose of pentobarbital and their brains were rapidly collected. The GFP^+^ area was dissected out with a 1 mm diameter punch, snap frozen in liquid nitrogen and stored at − 80 °C until protein extraction. Samples were homogenized by sonication in lysis buffer [50 mM Tris-HCl pH = 7.4, 150 mM NaCl, 1% Triton X-100 with 1:100 phosphatase inhibitors (Sigma, cocktail 2) and 1X protease inhibitors (Roche, Basel, Switzerland); 25 μl/punch] centrifuged at 20,000 g for 20 min at 4 °C. The supernatant contains Triton X-100-soluble proteins and was used for western blotting and MSD® ELISA tests.

### Western blot

Protein concentration was measured by the BCA test (Pierce, Waltham, MA). Samples were diluted in loading buffer with DTT (NuPAGE® LDS sample buffer and sample reducing agent, Invitrogen). Ten μg of proteins was loaded on a NuPAGE™ 4–12% Bis-Tris Midi Gel (Life Technologies). Migration was performed at 200 V for 45 min in NuPAGE™ Running Buffer (Invitrogen) and proteins were transferred on a nitrocellulose membrane with an iBlot Gel transfer device (Invitrogen). After 3 × 10 min rinses in Tris buffer saline and 0.1% Tween 20 (TBST), membranes were blocked in 5% milk in TBST for 1 h at RT and incubated for 3 h at RT, or overnight at 4 °C with the following primary antibodies: 6E10 (human APP, 1:500, Mouse, Covance, Princeton, NJ, #SIG-39320-20), anti-Actin (1:5,000, Mouse, Sigma, #A2066), anti-ApoE (1:1,000, Rabbit, Abcam, #ab20874), anti-BACE1 (1:1,000, Rabbit, Cell signaling, #5606P), anti-GFAP (1:5,000, Rabbit, Dako), anti-IDE (1:400, Rabbit, Abcam, #ab32216), and anti-Tubulin α (1:1,000, Mouse, Sigma, #T5168). After 3 x 10 min washes in TBST, membranes were incubated for 1 h at RT with HRP-conjugated secondary antibodies (1:5,000, Vector laboratories) diluted in TBST with 5% milk. Membranes were incubated with the Clarity Western ECL substrate (Bio-Rad) and the signal was detected with a Fusion FX7 camera (ThermoFisher Scientific). Band intensity was quantified with Image J and normalized to actin or tubulin α. Each antibody was used on at least 2 different membranes.

### MSD® ELISA tests

Triton X-100 soluble proteins were diluted in the diluent provided for the V-PLEX Aβ peptide panel kit (6E10 antibody, MSD®, Rockville, MD). Samples were loaded in triplicate and manufacturer’s protocol was followed. Aβ levels were quantified with the Discovery Workbench4.0, MSD® software thanks to a standard curve, and normalized to the protein content in each well.

### Cell sorting for RNAseq analysis

Mice were killed and their hippocampus rapidly collected in Hank’s Balanced Salt Solution (HBSS; Sigma). Cells were mechanically and enzymatically dissociated with fire-polished Pasteur pipettes and the neural tissue dissociation kit with papaïn (Miltenyi Biotec), following manufacturer’s instructions. After filtration through a 50 μm-filter, cells were centrifuged 10 min at 300 g, and diluted in 500 μl of HBSS. Myelin removal beads II and MS columns (Miltenyi Biotec) were used to deplete myelin from cell suspensions, as described by the manufacturer’s protocol. Cell sorting was performed on a BD Influx cell sorter, equipped with a 488 nm laser and a 530/40 detector. Non-fluorescent brain cells were used to set up the detector gain and position gates, which were kept constant throughout sorting. Cells were gated on a forward scatter/side scatter plot, then singlets were selected and finally cells were collected based on their GFP expression. Cells were centrifuged at 300 g for 5 min at RT, lyzed in 400 μl TRIzol (Invitrogen) and stored at − 80 °C before RNA extraction. The two hippocampi of each mouse was processed and sorted independently. Typically, 6,000 to 20,000 GFP^+^ astrocytes and 50,000 to 100,000 GFP^−^ cells were collected for each sample within 15–25 min.

### Measure of Aβ uptake in vivo by flow cytometry

Three hours before sacrifice, mice received an *i.p.* injection of 10 mg/kg MXO4 (diluted at 2.5 mg/ml in 50% DMSO in saline, pH = 12) to label amyloid material and monitor its uptake in cells [39]. Mice were perfused for 4 min with cold dPBS. Their hippocampus was rapidly collected in HBSS. Cells were dissociated and myelin was depleted as describe above. Cells were centrifuged at 300 g for 5 min at 4 °C and resuspended in Fc block (1: 100, TruStain FcX™, anti-mouse CD16/32, Biolegend, San Diego, CA) in HBSS for 10 min on ice. Samples were centrifuged at 300 g for 5 min at 4 °C and resuspended in cell staining buffer (Biolegend) with anti-CD11b-PE (1: 100, eBioscience, San Diego, CA) and anti-CD45-PE-Cy5 (1: 300, eBioscience) for 30 min on ice, under mild agitation. Cells were centrifuged at 300 g for 5 min at 4 °C and resupended in 400 μl HBSS. Cell sorting was performed on a BD Influx cell sorter. MXO4 was detected at 450/30 nm (408 nm excitation), GFP at 530/40 nm (488 nm excitation), CD11b-PE and CD45-PECy5 at 594/26 nm and 670/30 nm, respectively (561 nm excitation). MXO4^+^ and MXO4^−^ microglial cells were sorted separately and analyzed by RT-qPCR. Control samples of unlabeled or mono-fluorescent brain cells were used to set up detector gains and position sort gates, which were kept constant for all sorted samples. No compensation was required to accurately quantify MXO4, GFP, CD11b-PE ad CD45-PECy5 signals within the same sample.

### RNA extraction

Sorted cells were placed 5 min at RT and chloroform was added to TRIzol for 3 min. Samples were centrifuged at 12,000 g for 15 min at RT. Aqueous phase was collected and 1 volume of 70% ethanol was added. Samples were transferred onto an RNeasyMin Elute spin column and RNA was purified according to manufacturer’s instructions, with on-column DNAse treatment (RNeasy micro kit, Qiagen, Hilden, Germany). RNA was eluted with 14 μl of RNAse-free deionized water and stored at − 80 °C until transcriptomic analysis.

### RNAseq analysis

RNA quality and integrity were evaluated with an Agilent RNA 6000 Pico assay and the Agilent 2100 Bioanalyzer (Agilent technologies, Santa Clara, CA). As the number of sorted cells was limited, especially for GFP^+^ astrocytes, we quantified total RNA with Agilent RNA 6000 Pico assay and used 0.8 to 1 ng of total RNA per sample. Full length double strand cDNA libraries were constructed with the Smarter Ultra Low Input RNA kit v4 (Takara-Clontech, Mountain View, CA) with 11 to 14 LD-PCR cycles. Purification was done with Ampure XP (Beckman Coulter Genomics, Brea, CA). The full length ds cDNA libraries were qualified with an Agilent High Sensitivity DNA kit and quantified with the Qubit dsDNA HS Assay Kit (Invitrogen). All libraries were normalized to 750 pg in 5 μl as starting material to Nextera XT sample preparation kit (Illumina Incorporated, San Diego, CA). Ligation products were amplified with 12 PCR cycles. Stringent purification was done (0.6X of AMPure XP) to remove small inserts that would be sequenced preferentially. mRNAseq libraries were qualified and quantified with Agilent High Sensitivity DNA kit and Qubit dsDNA HS Assay Kit respectively, before sequencing on a HiSeq 2000 Illumina platform (2 × 100 bp). Quality controls and data analysis was performed by GenoSplice technology® (www.genosplice.com). Sequencing data quality, read repartition and insert size estimation were performed with FastQC, Picard-Tools, Samtools and rseqc. Reads were mapped on the mm10 Mouse genome assembly with STARv2.4.0 [17]. Gene expression analysis was performed as described previously [57]. Briefly, for each gene present in the Mouse FAST DB v2016_1_full annotations, reads aligning on constitutive regions (i.e. that are not prone to alternative splicing) were counted. Normalization of read counts and differential gene expression were performed with DESeq2 [49] on R (v.3.2.5). Only genes expressed in at least one of the two compared experimental conditions were analyzed. Genes were considered as expressed if their rpkm value was greater than 97.5% of the background rpkm value measured in intergenic regions. Results were considered statistically significant for *p* values ≤0.05 and fold-changes ≥1.5.

Three sets of analysis were performed: (1) 7 GFP^+^ astrocyte samples were compared to 3 GFP^−^ cell samples from the WT-GFP group, to validate sorting efficiency; (2) 4 GFP^+^ astrocyte samples from APP-GFP mice were compared to 7 GFP^+^ astrocyte samples from WT-GFP mice to identify transcriptional changes in reactive astrocytes due to AD; and (3) 5 GFP^+^ astrocyte samples from APP-SOCS3 mice were compared to 4 GFP^+^ astrocyte samples from APP-GFP mice to identify astrocyte genes that are normalized by SOCS3 in AD mice. Hierarchical clustering between GFP^+^ and GFP^−^ cell samples was generated with MeV software, using Euclidean distance and average agglomeration method. RNAseq datasets are deposited on GEO under reference GSE108520.

### Gene ontology analysis

Analysis for enriched Gene ontology (GO) terms were performed with Database for Annotation, Visualization and Integrated Discovery (DAVID) Functional annotation Tool (v6.8) [28] by Genosplice Technology®. GO terms and pathways were considered as enriched for fold enrichment ≥2.0, uncorrected *p* value ≤0.05 and minimum number of regulated genes in pathway/term ≥2.0.

### Weighted correlation network analysis (WGCNA)

WGCNA was performed with WGCNA R package [40] by Genosplice Technology®. Genes expressed in at least 20% of samples (14 029) were used. Modules with at least 30 genes and a kME > 0.7 were defined, with split = 2. ANOVA was performed on the Eigengene calculated in each sample, as defined in [40] and the module with a *p* < 0.05 was further analyzed by STRING to identify functional networks of regulated genes (https://string-db.org/).

### RT-qPCR

RT-qPCR were performed on sorted astrocytes or microglial cells. Reverse transcription was performed with the VILO™ kit according to the manufacturer’s protocol (SuperScript® VILO™ cDNA synthesis kit; Life Technologies, Carlsbag, CA). Samples were diluted at 0.2 ng/μl in H_2_O with 100 μg/ml BSA and mixed with 250 nM of primers and Platinum SYBR-Green® (Platinum® SYBR® Green qPCR SuperMix-UDG; Life Technologies) for qPCR. PCR efficiency was between 85 and 110% for each set of primers (sequences shown in Additional file 2: Table S1). Nuclease-free water and samples without reverse transcription were used as negative controls. Expression levels of transcripts of interest were normalized with the ∆Ct method to the abundance of *Rpl13a* and *actin* for hippocampal astrocytes or *Eef1a1* and *Actin* for microglial cells.

### Electrophysiological recordings

Transverse hippocampal slices were prepared as described previously [65], from 8 to 9 month-old WT-GFP, 3xTg-GFP and 3xTg-SOCS3 mice and from 4 to 6 month-old WT-GFP and WT-JAK2ca mice. Briefly, mice were anesthetized with 5% isoflurane and decapitated. The brain was rapidly removed and placed in ice-cold artificial cerebrospinal fluid (ACSF) saturated with 95% O_2_ and 5% CO_2,_ containing (in mM): 125 NaCl, 2.5 KCl, 1.25 NaH_2_PO_4_, 1.3 MgCl_2_, 2 CaCl_2_, 26 NaHCO_3_ and 10 glucose (pH = 7.4; 305 mOsmol/kg). A block of tissue containing the hippocampus was prepared and 350 μm transversal hippocampal slices were cut on a vibratome (Leica). Slices were incubated 30 min at 32 °C and allowed to recover for at least 1 h at RT.

Slices were transferred to a recording immersion chamber and were perfused with ACSF (3 ml/min) at RT during the whole experiment. For the input/output experiment in WT and 3xTg mice, an incision between CA3 and CA1 regions was made in presence of 50 μM picrotoxin in the perfusion bath. CA3 and CA1 areas were identified with differential interference contrast microscopy and the region of interest (GFP^+^) was visualized with the epifluorescent mode of the microscope (Olympus BX50, Tokyo, Japan). Extracellular field excitatory postsynaptic potentials (fEPSPs) were evoked by orthodromic stimulations (100 μs, 0.033 Hz) of Schaffer collaterals with a glass pipette filled with ACSF or a tungsten bipolar electrode placed in the *stratum radiatum,* more than 150 μm away from the recording electrode. Then, fEPSPs were recorded, in current clamp mode (*I* = 0), with a glass pipette filled with ACSF (2–3 MΩ) and placed in the GFP^+^ region, in the *stratum radiatum* of CA1 area. Data were acquired with a Multiclamp 700B amplifier (Molecular Devices, Sunnyvale, CA), digitized with a Digidata 1320A digitizer (Axon Instruments Inc., Sunnyvale, CA), recorded and analyzed off line with pClamp and Clampfit 10.3 respectively (Molecular Devices). Recordings were low-pass filtered at 2 kHz and digitized at 10 kHz.

A stable baseline was recorded for at least 20 min before starting the input/output experiment. The same amplitude of stimulation was applied three times (0.033 Hz), before being increased by 10 V. Paired-pulse stimulation experiments were performed with two pulses of stimulation induced at 50 or 100 ms interval. The paired pulse ratio was calculated as the slope of the second fEPSP over the slope of the first fEPSP. Long-term potentiation (LTP) was induced after 20 min of baseline recording, by applying a high-frequency stimulation (HFS) protocol (100 Hz train of stimuli for 1 s, repeated three times at 20 s interval).

At the end of the recording session, slices were post-fixed in 4% paraformaldehyde for 48 h and processed for GFAP immunostaining following the protocol described above except that GFAP-Cy3 antibody was incubated at 1:200 for 48 h.

### Morris water maze

The water maze was a white circular pool (120 cm diameter, 60 cm high) filled with water and white soluble paint at 21 °C. The testing room contained numerous black extra maze cues and was illuminated with 400 Lux at the pool center. Mouse behavior (distance to the platform and swim speed) was monitored by a video camera, mounted on the ceiling above the pool center, and a computerized tracking system (Ethovision 11.5, Noldus IT, The Netherlands). All tests were performed between 8 am and 1 pm. Mice were handled daily for 2 min for 5 d preceding the training. First, their ability to see and shelter on an emerged platform (11 cm diameter) was assessed with four trials of 60 s in one day, with an intertrial interval (ITI) of 30 min, each with a different platform location. A 10 cm high object was placed on the emerged platform. For the training phase, mice were given three trials every day for 5 d, with an ITI of 30 min. The starting position differed for each trial but was identical for all animals, with mice placed in the water facing the wall of the pool. An invisible escape platform was placed in the middle of one of the quadrants (1 cm below the water surface) equidistant from the sidewalls and the middle of the pool. The platform location remained constant throughout training. Each trial lasted 60 s or until the animal located the platform. Animals that did not find the platform were guided to it and given a latency score of 60 s. All animals were left on the platform for 30 s and then taken back to their home cage before starting the next trial. 72 h after training, animals were given one 30 s probe trial during which the platform was removed from the pool. The probe trial started from a position opposite to the platform. The pool was divided into four quadrants of equal size. The percentage of time spent in each quadrant was recorded. No mouse was excluded from the analysis.

### Statistics

Results are expressed as mean ± SEM. N are indicated for each group in the order presented on the histogram. No statistical method was used to predetermine sample size. Sample size was chosen based on prior experience for each experiment, to yield adequate power to detect specific effects. Statistical analysis were performed with Statistica software (StatSoft, Tulsa, OK) or GraphPad Prism 7 (La Jolla, CA). We used paired or unpaired two-tailed Student *t* test to compare two groups and repeated-measures or one-way ANOVA and Tukey’s post hoc test to compare three groups. For each analysis, normality of residues and homoscedasticity were assessed, as well as sphericity for repeated-measures ANOVA. If any conditions of application was not fulfilled, we used non-parametric tests. Two groups were compared by the Mann-Whitney or Wilcoxon test and three independent groups were compared by a Kruskal-Wallis test followed by post-hoc comparison of mean ranks. Percentages were normalized by Arcsin transformation and analyzed with parametric tests. The significance level was set at *p* < 0.05. Investigators were partially blinded to the group when performing experiments and measuring outcomes (as the group can be guessed based on the presence of amyloid plaques or GFP levels for example).

## Results

### The JAK2-STAT3 pathway controls astrocyte reactivity in AD mice

To study whether the JAK2-STAT3 pathway controls astrocyte reactivity in AD, we inhibited this pathway by overexpressing its endogenous inhibitor Suppressor Of Cytokine Signaling 3 (SOCS3, [6]) in APP/PS1dE9 mice (hereafter called APP mice). We used adeno-associated vectors (AAV) that target astrocytes (Additional file 1: Figure S1) to express SOCS3 or GFP in hippocampal astrocytes. APP mice display progressive astrocyte reactivity in the hippocampus, where amyloid plaques accumulate [8, 30]. They were injected with AAVs in the CA1 region of the hippocampus, before detectable amyloid plaque deposition. AAV-SOCS3 was co-injected with AAV-GFP to visualize infected astrocytes (APP-SOCS3 mice). Control mice included APP and WT littermates injected with AAV-GFP alone, at the same total viral titer (Fig. 1a). Approximately 25% of the hippocampus was targeted by these AAV, as quantified with GFP staining, and more than 97% of GFAP^+^ astrocytes were GFP^+^ within the infected volume (data not shown). By immunostaining, we found that nuclear STAT3 levels in hippocampal astrocytes were higher in 9 month-old APP mice than in WT-GFP mice, indicating STAT3 activation. STAT3 immunoreactivity was efficiently reduced by SOCS3 in APP mice at 9 months (Fig. 1b, c) and even more so at 12 months (Additional file 3: Figure S2). Of the seven members of the STAT family, astrocytes express STAT3 at highest levels ([75] and our own RNAseq data). However, STAT1 was proposed to play important roles in brain inflammatory processes [71]. Here, we found that STAT1 expression was not induced in APP mice, confirming that STAT3 is the major STAT effector in astrocytes (Additional file 3: Figure S2). Likewise, extracellular signal-regulated kinase (ERK), a kinase that could also be inhibited by SOCS3 [36] was not activated in APP astrocytes and not further modulated by SOCS3 (Additional file 3: Figure S2).

Compared to WT, APP astrocytes were hypertrophic and overexpressed GFAP, the two classical hallmarks of reactive astrocytes (Fig. 1b, d). SOCS3 prevented GFAP upregulation in the hippocampus of APP mice, as shown by immunostaining (Fig. 1b, d) and western blotting on hippocampal samples from WT-GFP, APP-GFP and APP-SOCS3 mice (Fig. 1f, g). SOCS3 also reduced the number of vimentin^+^ astrocytes, another intermediate filament upregulated in reactive astrocytes (Fig. 1e).

Interestingly, over-activation of STAT3 by expression of a constitutively active mutant of the upstream kinase JAK2 (JAK2ca [23]) was able to further increase astrocyte reactivity in APP mice. By contrast to APP-GFP mice, APP-JAK2ca mice displayed enhanced STAT3 nuclear accumulation in astrocytes (Fig. 1b, c) and increased GFAP and vimentin protein levels (Fig. 1b-g).

Importantly, SOCS3 was also efficient in aged APP mice that already display strong astrocyte reactivity. Injection of AAV-SOCS3 in the hippocampus of 15 month-old APP mice reversed established astrocyte reactivity, as seen by a significant decrease in GFAP levels (Fig. 1h, i**)**. These results show that the JAK2-STAT3 pathway is necessary for the induction and long-term maintenance of the basic morphological features of reactive astrocytes in APP mice.

### SOCS3 normalizes multiple reactive astrocyte markers in AD mice

**Fig. 2:**
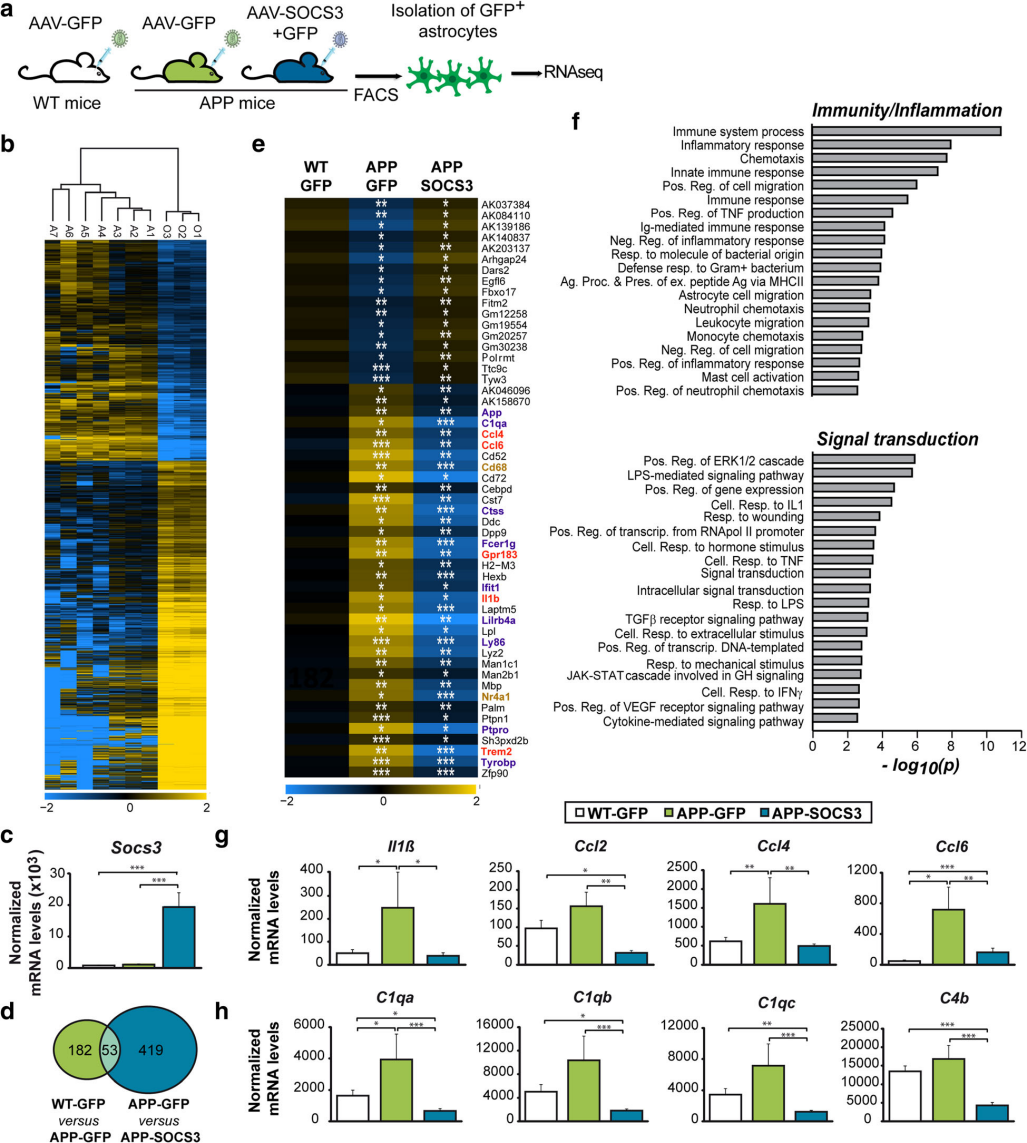
SOCS3 restores the transcriptional profile of APP astrocytes. **a**, Hippocampal astrocytes of 9 month-old WT-GFP (*N* = 7), APP-GFP (*N* = 4) or APP-SOCS3 (*N* = 5) mice were isolated by FACS and their transcriptome examined by RNAseq analysis. **b**, Hierarchical clustering of the ~ 7000 differentially expressed genes between GFP^+^ astrocytes (samples A1-A7) and all other GFP^−^ cells (samples O1-O3), which comprise microglial cells, neurons, oligodendrocyte precursor cells and non-infected astrocytes. **c**, *Socs3* mRNA levels are increased more than 10 times in APP-SOCS3 astrocytes compared to WT-GFP and APP-GFP astrocytes. **d**, Venn Diagram showing the number of differentially expressed genes between WT-GFP and APP-GFP astrocytes and APP-GFP and APP-SOCS3 astrocytes. **e**, Expression levels for the 53 genes dysregulated in APP-GFP astrocytes and normalized in APP-SOCS3. Color scale represents mean-centered expression (log2-transformed). Genes belonging to immunity/inflammation pathways are in purple, those belonging to signal transduction are in brown. Genes common to the two pathways are in red. **f**, Pathway analysis on the 472 genes regulated by SOCS3 in APP astrocytes reveals a specific enrichment in GO terms linked to immunity/ inflammation and signal transduction. Ag. Proc & Pres. = antigen processing and presentation. Cell. = cellular. Ex. = exogenous. Neg. = negative. Pos. = positive. Reg. = regulation. Resp. = response. **g**, **h**, SOCS3 normalizes gene expression of cytokines/chemokines (**g**) and complement factors (**h**), which are induced in APP-GFP astrocytes. Wald test, * *p* < 0.05, ** *p <* 0.01, *** *p <* 0.001

Besides morphological alterations, astrocyte reactivity is characterized by significant gene expression changes, especially during AD [48, 62, 85]. We thus studied the effects of SOCS3 on the transcriptional profile of APP astrocytes. We performed RNA sequencing (RNAseq) of GFP^+^ astrocytes acutely isolated by FACS from WT-GFP, APP-GFP and APP-SOCS3 hippocampi (Fig. 2a). GFP^−^ cells comprising microglia, neurons, oligodendrocyte precursor cells and non-infected astrocytes were collected together. Genome-wide RNAseq analysis of GFP^+^ astrocytes and other GFP^−^ cells from WT-GFP mice identified 6949 genes differentially-expressed (Fold change > 1.5 and *p* < 0.05, Fig. 2b). Expression of cell-type specific markers validated the purity of sorted GFP^+^ astrocytes (Additional file 4: Figure S3).

After validation of sorting efficiency, we then compared the transcriptome of GFP^+^ astrocytes between the three groups. *Socs3* mRNA levels were 12 to 16 times higher in astrocytes of the APP-SOCS3 group than in the APP-GFP and WT-GFP groups respectively (Fig. 2c). We found hundreds of genes differentially expressed between APP-GFP and WT-GFP astrocytes groups and between APP-SOCS3 and APP-GFP astrocytes (Fig. 2d). A majority of genes (85%) were down-regulated by SOCS3, consistent with its inhibitory action on STAT3-mediated transcription. Several genes previously described as up-regulated in astrocytes acutely isolated from 15 month-old APP mice [62], were already induced at 9 months in APP-GFP compared to WT-GFP astrocytes (e.g. *Cst7, Ccl4, Ccl6, Ctss, Trem2, Tyrobp, Il1b*). Gene Ontology (GO) analysis with DAVID showed an enrichment in biological processes related to inflammation/immunity (data not shown), as reported in 15 month-old APP mice [62], as well as in laser captured cortical astrocytes from AD patients [73]. Fifty-three genes were either down- or up-regulated in APP-GFP mice relatively to WT-GFP mice and restored by SOCS3 (Fig. 2d, e). This list was also significantly enriched in GO biological processes linked to inflammation/immunity and signal transduction (*p* < 0.05, Fig. 2e). In the list of 472 genes regulated by SOCS3 in APP mice, there was an even larger number of enriched GO terms linked to these biological processes (*p* < 3.10^− 3^, Fig. 2f). For example, SOCS3 abrogated the induction of the cytokines and chemokines *Il1β, Ccl2, Ccl4* and *Ccl6* observed in APP-GFP astrocytes (Fig. 2g). Interestingly, several complement factors (*C1qa, C1qb, C4b, C1qc),* were down-regulated by SOCS3 in APP astrocytes (Fig. 2h).

**Fig. 3:**
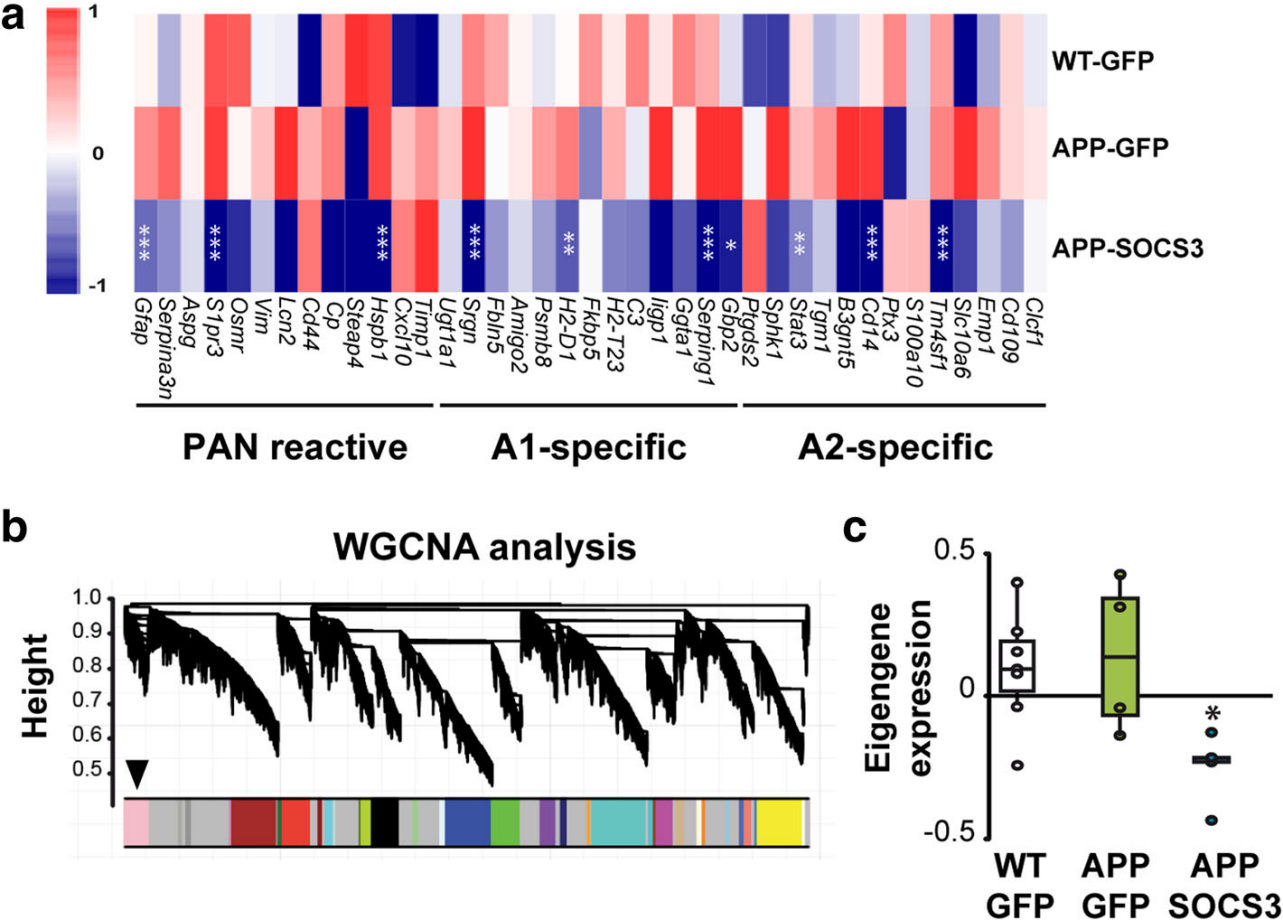
SOCS3 inhibits the expression of reactive astrocyte markers. **a**, Heatmaps of genes belonging to the pan, A1 or A2 reactive astrocyte cassettes. SOCS3 decreases the expression of markers belonging to all categories, in APP astrocytes. Color scales represent mean-centered expression (log2-transformed). Wald test. **b**, Dendrogram obtained by WGCNA with the significant module indicated with an arrow. **c**, The significant WGCNA module is mainly formed by genes down-regulated by SOCS3. ANOVA. *N* = 7-4-5. * *p* < 0.05, ** *p <* 0.01, *** *p <* 0.001

We then studied SOCS3 effects on reactive astrocyte genes, as described in previous transcriptional studies [14, 48]. Strikingly, SOCS3 reduced the expression of genes from all three categories of reactive markers (pan, A1 and A2) that were induced in APP astrocytes (Fig. 3a). Few genes were not down-regulated by SOCS3, suggesting that even if SOCS3-expresing astrocytes show a significant down-regulation of reactive genes, they may still not be fully comparable to normal astrocytes from WT mice. Last, to gain insight into the transcriptional networks regulated by SOCS3 in APP astrocytes, we performed a weighted gene correlation network analysis (WGCNA). This statistical method identifies modules of co-regulated genes across samples. One module among 19 was identified as differentially expressed between the three groups (*p* = 0.018, Fig. 3b). This module was comprised of 567 genes mainly down-regulated by SOCS3 (Fig. 3c). Interestingly, this module contained several pan *(Gfap, Vimentin)*, A1 (*Serping1*, *H2-D1, Srgn)* and A2 (*Tm4sf1, CD14*) genes (Additional file 5: Table S2), emphasizing that markers of these two extreme classes are co-regulated by SOCS3. This module also contained genes related to inflammation (e.g. *C1qa, C1qb, C1qc, Ccl3,* Additional file 5: Table S2, Additional file 6: Figure S4). We performed an interaction network analysis with STRING on the 100 most connected genes of the module. Gene networks linked to complement system/inflammation were identified, as well as cytoskeleton and cell adhesion, which may underlie the morphological changes characteristic of reactive astrocytes (Additional file 6: Figure S4).

Overall, these results show that SOCS3 operates as a master inhibitor on transcriptional programs of reactivity, regulating different neuroinflammatory markers in reactive astrocytes.

#### JAK2-STAT3-mediated astrocyte reactivity promotes amyloid deposition in APP mice

**Fig. 4:**
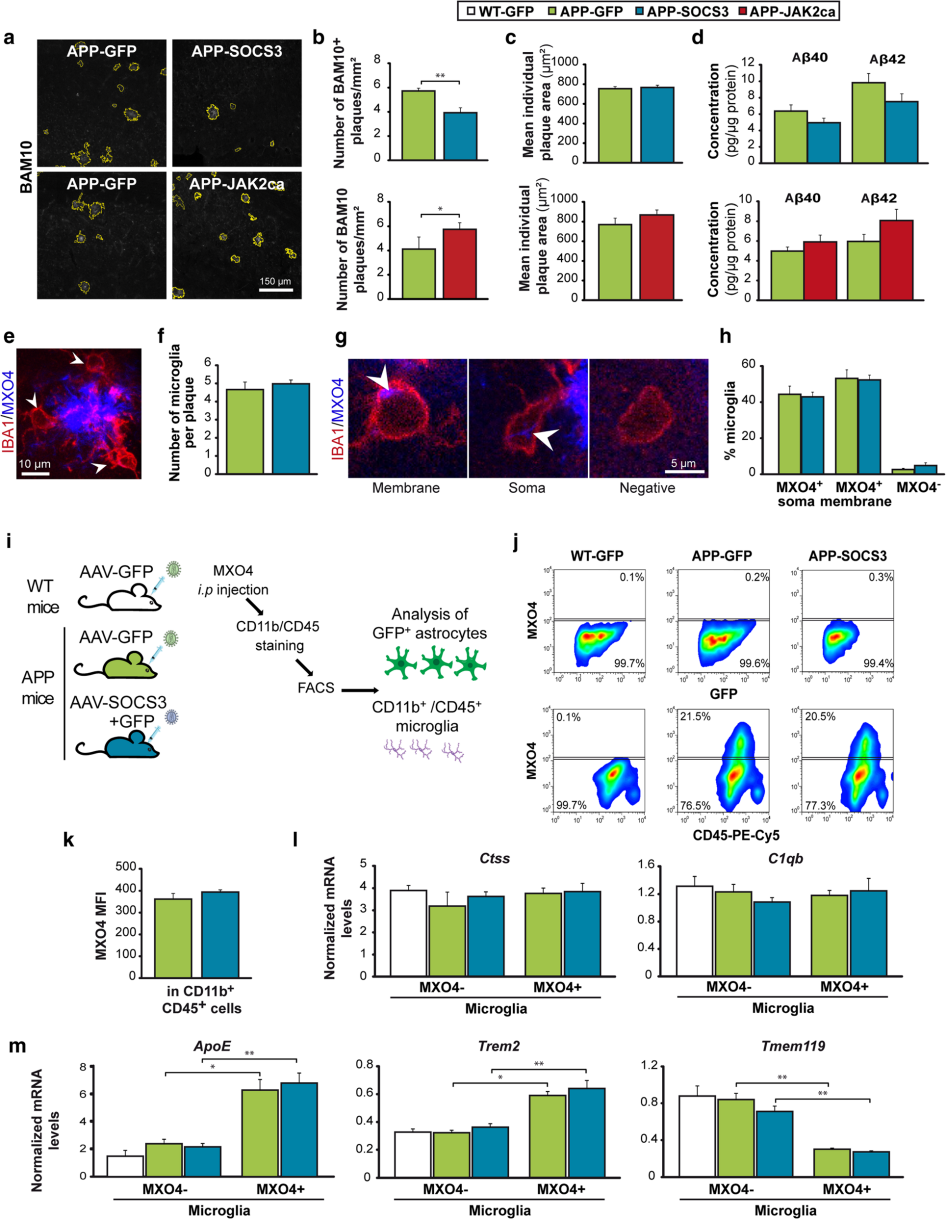
Inhibition of JAK2-STAT3-mediated astrocyte reactivity reduces amyloid load in APP mice without impacting microglial cells **a**, Representative images of BAM10^+^ amyloid plaques (white) automatically delineated in yellow in APP-GFP, APP-SOCS3 and APP-JAK2ca mice. **b**, The number of hippocampal BAM10^+^ plaques is significantly decreased by SOCS3 (*N* = 9–8) and increased by JAK2ca in APP mice (*N* = 6–6). SOCS3 and JAK2ca effects were measured in two independent cohorts. **c**, The average size of individual BAM10^+^ amyloid plaque is similar between groups. **d**, Dosage of Aβ40 and Aβ42 peptide concentrations in Triton-X100-soluble protein homogenates from the hippocampus of APP-GFP (*N* = 10 or 6), APP-SOCS3 (*N* = 8) and APP-JAK2ca mice (*N* = 6). Aβ40 and 42 levels are not significantly different between groups. **e**, IBA1^+^ microglial cells (red, arrowheads) in contact with a MXO4^+^ amyloid plaque (blue). **f**, The number of microglia per plaque is similar between APP-GFP and APP-SOCS3 mice. *N* = 10–8. **g**, Confocal images of MXO4^+^ material (blue) in IBA1^+^ microglial cells (red). Microglial cells in contact with plaques either display MXO4 staining (white arrowhead) at the membrane, in the cytosol or are MXO4^−^. **h**, The proportion of these three classes of microglial cells is not different between groups. *N* = 10–8. **i**, Experimental design to monitor Aβ phagocytosis. WT-GFP (*N* = 10), APP-GFP (*N* = 6) and APP-SOCS3 (*N* = 8) mice were injected with MXO4, 3 h before sacrifice. After staining, hippocampal CD11b^+^/CD45^+^ microglia and GFP^+^ astrocytes were analyzed by FACS. **j**, Representative gates to analyze MXO4^+^ amyloid uptake in astrocytes and microglia. There are 20% MXO4^+^ microglial cells in both APP-GFP and APP-SOCS3 groups and no MXO4^+^ astrocytes. **k**, No difference in the MXO4 median fluorescent intensity (MFI) is observed between APP-GFP and APP-SOCS3 microglial cells. **l**-**m**, RT-qPCR analysis on microglial cells acutely isolated from the hippocampus of 12 month-old WT-GFP, APP-GFP and APP-SOCS3 mice. **l** mRNA levels of *Ctss* and *C1qb,* two microglial homeostatic genes, is similar in all groups. **m**, *Apoe* and *Trem2* mRNA levels are higher in phagocytic MXO4^+^ microglia than non-phagocytic MXO4^−^ microglia, while *Tmem119* levels are lower in MXO4^+^ microglia. This transcriptional profile is reminiscent of DAM microglia [34]. Astrocyte de-activation by SOCS3 does not impact the transcriptional profile of either type of microglia. *N* = 3–8/group. **b**, **d**, **h**, Student t test. **c**, **f**, **k**, Kruskall-Wallis test. **l**, **m**, One way ANOVA to compare the 3 groups within MXO4^−^ cells and Student t test to compare two groups within MXO4^+^ cells. Mann-Whitney test to compare MXO4^+^ and MXO4^−^ microglial cells within APP-GFP or APP-SOCS3 groups. * *p* < 0.05, ** *p* < 0.01

The identification of a master regulator of reactive astrocytes makes it possible to evaluate their overall contribution to AD pathological outcomes. We first investigated the impact of JAK2-STAT3-mediated astrocyte reactivity on amyloid deposition, a major histopathological hallmark of AD [24]. SOCS3-mediated inhibition of astrocyte reactivity significantly reduced the number of BAM10^+^ amyloid plaques in the hippocampus of 9 month-old APP mice (Fig. 4a, b). This effect was also observed after labeling plaques with methoxy-XO4 (MXO4), a fluorescent Congo red-derivative that binds aggregated amyloid (− 36%, *p* < 0.05, Student *t* test, data not shown). Importantly, over-activation of astrocytes with JAK2ca had opposite effects (Fig. 4a, b). The average size of individual plaques was identical between groups, suggesting that only the number, not the properties of plaques was impacted by SOCS3 (Fig. 4c). Levels of soluble human amyloid β (Aβ)42 and Aβ40 peptides, and their ratio were not significantly different between APP-GFP, APP-SOCS3 or APP-JAK2ca mice (Fig. 4d). Moreover, changes in amyloid plaque load were not due to changes in the expression of proteins involved in amyloid precursor protein (APP) metabolism. Indeed, protein levels of APP itself, of the pro-amyloïdogenic β-secretase BACE1, or of insulin degrading enzyme (IDE) and apolipoprotein E (ApoE), two proteins released by astrocytes and involved in Aβ elimination, were not impacted by SOCS3 or JAK2ca (Additional file 7: Figure S5).

We then focused on SOCS3, as its plaque lowering effects were more therapeutically relevant. Because microglia play a key role in amyloid plaque elimination via phagocytosis [42], we investigated whether inhibition of astrocyte reactivity increases microglia phagocytic activity, which could explain decreased amyloid load in APP-SOCS3 mice. The number of IBA1^+^ microglial cells in contact with MXO4^+^ amyloid plaques was not different between APP-SOCS3 and APP-GFP mice (Fig. 4e, f). MXO4 also labelled intracellular amyloid material in IBA1^+^ microglial cells on brain sections. In fact, more than 95% of microglia cells in contact with plaques were MXO4^+^, revealing active microglial phagocytosis of amyloid plaques (Fig. 4g, h). MXO4^+^ material was either localized at the membrane or within the soma (Fig. 4g), but the relative localization of MXO4^+^ within microglia was not different between APP-SOCS3 and APP-GFP mice (Fig. 4h). To have dynamic quantification of microglia (and astrocyte) phagocytic capacity, MXO4 was injected *i.p.* 3 h before mouse sacrifice and its accumulation within dissociated brain cells was monitored by FACS. Astrocytes were identified by their GFP expression, while microglial cells were labelled with CD11b and CD45 antibodies (Fig. 4i). The percentages of GFP^+^ astrocytes (4%) and total microglial cells (14%) were not different between WT-GFP, APP-GFP and APP-SOCS3 mice (data not shown). No MXO4 uptake was detected in WT cells (Fig. 4j). Strikingly, we found no evidence for active MXO4 uptake in GFP^+^ astrocytes (Fig. 4j). In addition, the expression of several receptors or proteins involved in phagocytosis (*Abca1, Apoe, Axl, Gulp1, Itgav, Itgb5, Ldlr, Lrp1, Megf10, Mertk,* [10, 55]) were expressed at similar levels in astrocytes of the three groups or only down-regulated by SOCS3 (*Fcer1g, Fcgr2b* and *Fcgr3,* Additional file 8: Table S3), further suggesting that astrocyte phagocytosis is not involved in reduced amyloid deposition with SOCS3. In contrast to astrocytes, ~ 20% of CD11b^+^/CD45^+^ microglial cells accumulated MXO4^+^ material in both APP-GFP and APP-SOCS3 mice (Fig. 4j). MXO4 median fluorescent intensity in microglia was not different between these two groups (Fig. 4k). A sub-class of microglia with active phagocytic capacities was recently described as « disease-associated microglia » DAM, [34]. It is possible that inhibition of astrocyte reactivity with SOCS3 only impacts a specific class of microglial cells like DAM. To explore this possibility, expression of genes specific for homeostatic microglia (*Ctss* and *C1qb*) and DAM cells were analyzed in MXO4^−^ and MXO4^+^ microglial cells. MXO4^+^ microglia had a gene profile characteristic of DAM (up-regulation of *ApoE* and *Trem2*; downregulation of *Tmem119*), consistent with the strong phagocytic activity of these cells. SOCS3 expression in astrocytes did not change the levels of homeostatic microglia or DAM-specific markers (Fig. 4l, m).

Overall, we show that SOCS3 does not significantly impact microglial molecular profile or phagocytic capacity. The reduction in amyloid deposition with SOCS3 is not due to enhanced phagocytosis by glial cells.

#### Inhibition of astrocyte reactivity improves spatial learning in APP mice

**Fig. 5:**
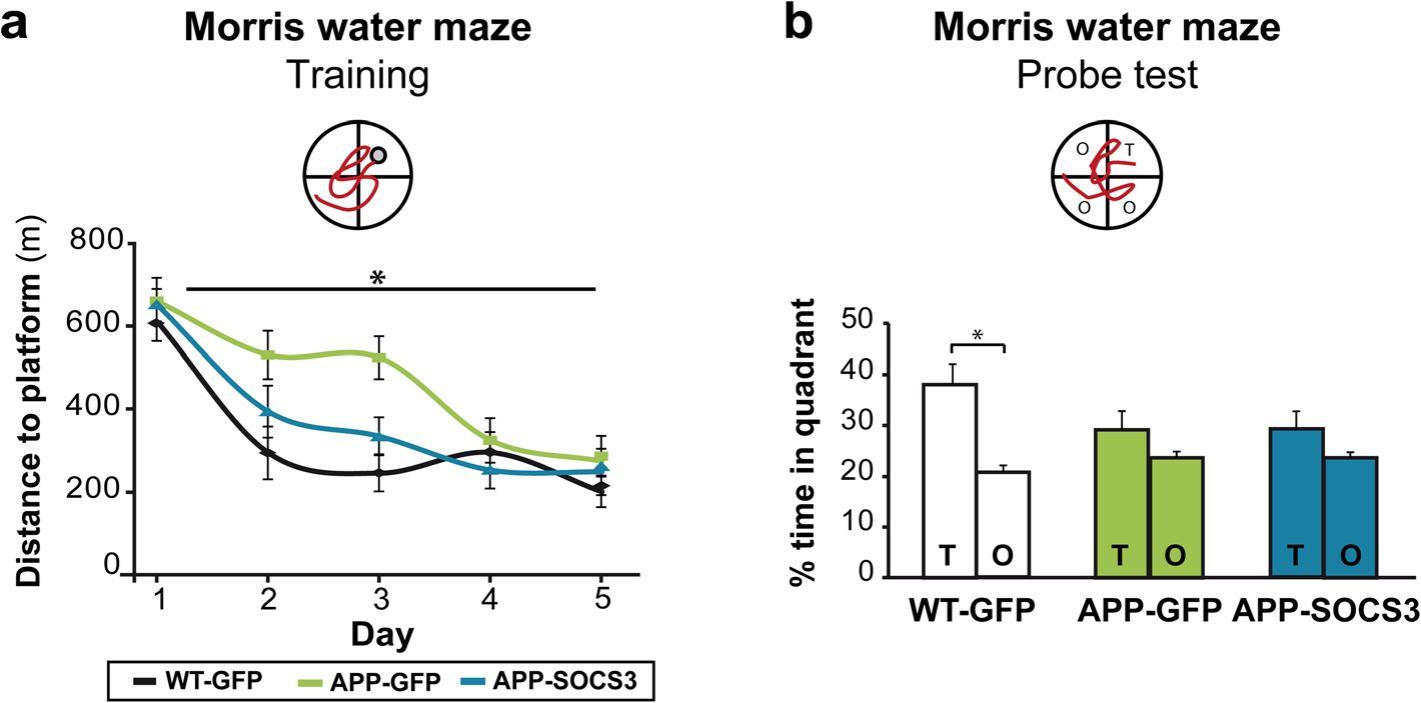
Inhibition of STAT3-mediated astrocyte reactivity improves spatial learning in APP mice **a**, Training phase of the Morris water maze. APP-GFP mice (*N* = 12) need more trials to learn the task than WT-GFP mice (*N* = 11). This learning deficit is corrected by SOCS3 expression in APP astrocytes (*N* = 11). Repeated-measures ANOVA. **b**, Probe phase of the Morris Water maze, 72 h after the last training session. Unlike WT-GFP mice, APP-GFP mice do not display preference for the target quadrant (T) over other quadrants (O). This memory deficit is not corrected by SOCS3. Wilcoxon test. * *p* < 0.05

We next tested whether SOCS3-mediated inhibition of astrocyte reactivity improved behavioral defects in APP mice. Nine month-old WT-GFP, APP-GFP and APP-SOCS3 mice were tested on the Morris water maze to assess spatial learning and memory. Swim speed was not different between groups (*p* = 0.63 for group effect, repeated-measure ANOVA). As previously described [16], APP-GFP mice displayed delayed task learning, as they only improved their ability to find the hidden platform by training day 4, while WT-GFP mice achieved this performance by day 2 (Fig. 5a, repeated-measures ANOVA *p* = 0.0136 for group effect, *p* < 10^− 6^ for day effect). By contrast, APP-SOCS3 mice learnt the task as quickly as WT-GFP mice, showing that SOCS3 corrects learning deficits in APP mice. Mice were probed for spatial memory 72 h after the last day of training. WT-GFP mice displayed the expected preference for the target quadrant, while APP-GFP mice explored all quadrants similarly (Fig. 5b). SOCS3 did not improve spatial memory in APP mice (Fig. 5b), showing that inhibition of hippocampal astrocyte reactivity by SOCS3 improves spatial learning but not memory retrieval in APP mice.

#### SOCS3 restores synaptic transmission and plasticity in 3xTg mice

**Fig. 6:**
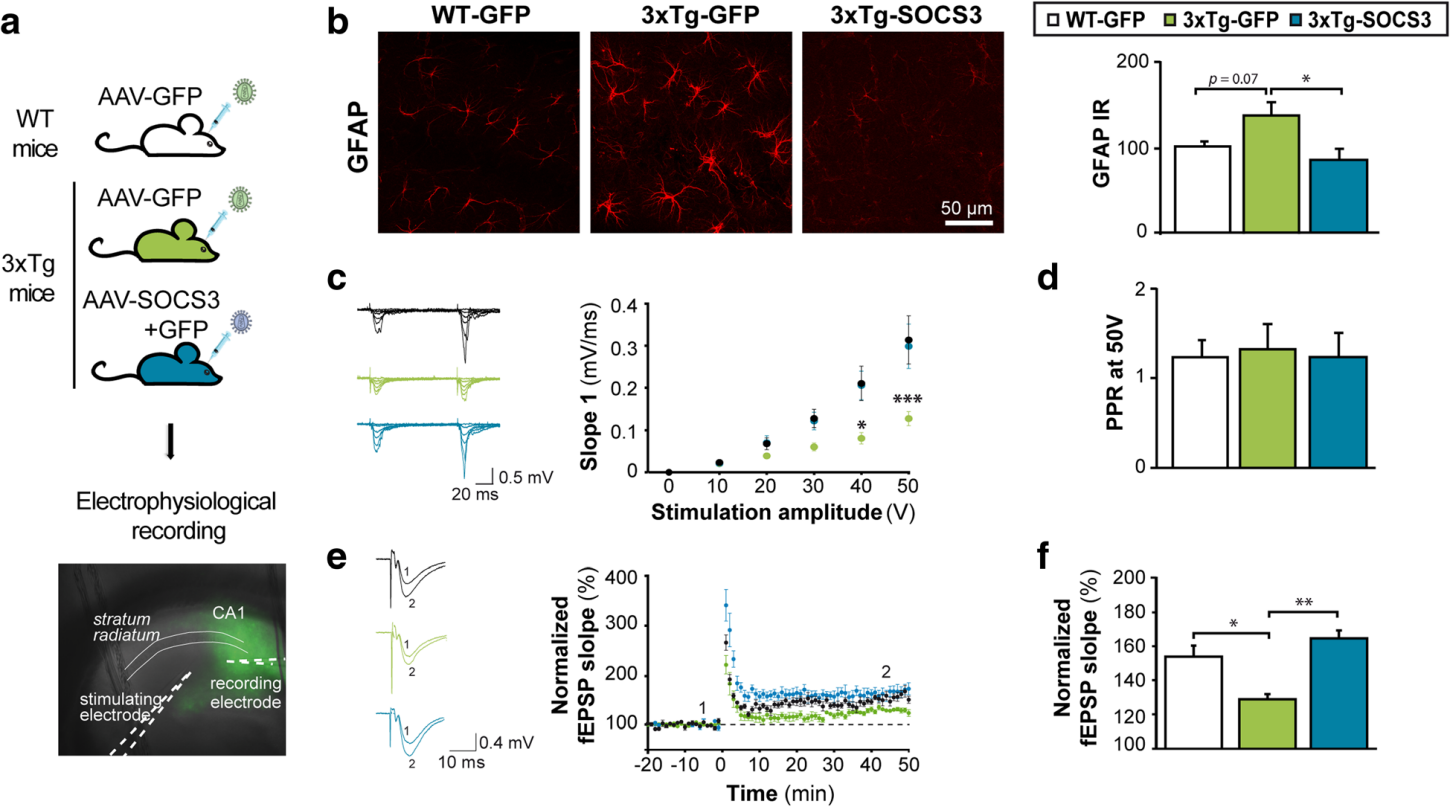
SOCS3 rescues synaptic transmission and long-term plasticity in the hippocampus of 3xTg mice. **a**, Acute hippocampal slices were prepared from the hippocampus of 8–9 month-old WT-GFP, 3xTg-GFP and 3xTg-SOCS3 mice. A recording electrode was placed in the *stratum radiatum* of the GFP^+^ CA1 region. **b**, Acute slices processed for GFAP immunohistochemistry (red). In 3xTg-GFP mice, astrocytes display higher GFAP immunoreactivity and tortuous processes, compared to WT-GFP controls. SOCS3 restores low GFAP levels in 3xTg astrocytes *N* = 8–7-6. **c**, Representative traces for WT-GFP, 3xTg-GFP and 3xTg-SOCS3 mice after a paired-pulse stimulation protocol (50 ms interval) with increasing voltage. The input/output relationship is impaired in 3xTg-GFP mice and restored by SOCS3. *N* = 11-7-7. Two way (group, voltage) ANOVA and Tukey’s test. **d**, The paired-pulse ratio (PPR) at 50 V is similar in the three groups. *N* = 11-7-7. ANOVA. **e**, Representative (left) and average (right) fEPSPs before (1) and after (2) HFS protocol in the three groups. LTP is impaired in 3xTg mice and restored by SOCS3. **f**, Normalized fEPSP slopes 40 to 50 min post HFS, relatively to fEPSPs measured 10 min before HFS. *N* = 6-6-5. ANOVA and Tukey’s test. * *p* < 0.05, ** *p* < 0.01, *** *p* < 0.001

Given that astrocyte reactivity inhibition restores a complex behavioral task, we then examined the underlying cellular mechanisms in acute hippocampal slices. We studied synaptic transmission and plasticity, which are altered in AD [74] and regulated by astrocytes [5]. For this study, we used 3xTg-AD mice (thereafter called 3xTg mice) that display more robust synaptic deficits than APP mice [50]. 3xTg mice show early impairment in synaptic transmission and long-term plasticity before amyloid and Tau pathology [59]. 3xTg mice were injected at 3–4 months with AAV-GFP or AAV-SOCS3 + AAV-GFP (3xTg-GFP and 3xTg-SOCS3 mice respectively) and studied 5 months later, when they display synaptic deficits and higher GFAP levels [11, 53] but before amyloid plaque and Tau deposition ([59], Fig. 6a). Like in APP mice, SOCS3 was able to block astrocyte reactivity in the hippocampus of 3xTg mice, as seen by significantly lower GFAP levels in 3xTg-SOCS3 mice than in 3xTg-GFP mice (Fig. 6b). We then studied basal glutamatergic synaptic transmission, short-term and long-term synaptic plasticity in acute hippocampal slices prepared from the three groups. Field excitatory post-synaptic potentials (fEPSPs) were recorded in the infected, GFP^+^ region of the *stratum radiatum* in the CA1 area (Fig. 6a). As reported previously in 3xTg-GFP mice [59], the input-output relationship for evoked fEPSPs at CA3-CA1 synapses was shifted to the right, indicating impaired basal glutamatergic synaptic transmission (Fig. 6c). This was probably not due to a modification of glutamate release probability because the paired-pulse ratio was unchanged (Fig. 6c, d). Interestingly, inhibition of astrocyte reactivity by SOCS3 rescued the input-output relationship (Fig. 6c). As reported before [59], LTP induced by high frequency stimulation (HFS) of Schaffer collaterals was impaired in 3xTg-GFP mice, in comparison to WT-GFP mice that displayed a 50% increase in fEPSPs after HFS (Fig. 6e, f). Strikingly, SOCS3 fully restored LTP deficits in 3xTg mice (Fig. 6e, f), suggesting that astrocyte reactivity plays a major role in AD-related synaptic alterations, and that these alterations can be reversed.

### JAK2-STAT3 pathway activation in astrocytes induces reactivity and synaptic alterations

We reasoned that if synaptic deficits in 3xTg mice are due to JAK2-STAT3 pathway activation in astrocytes, its stimulation in naïve WT mice should result in comparable synaptic deficits.

**Fig. 7:**
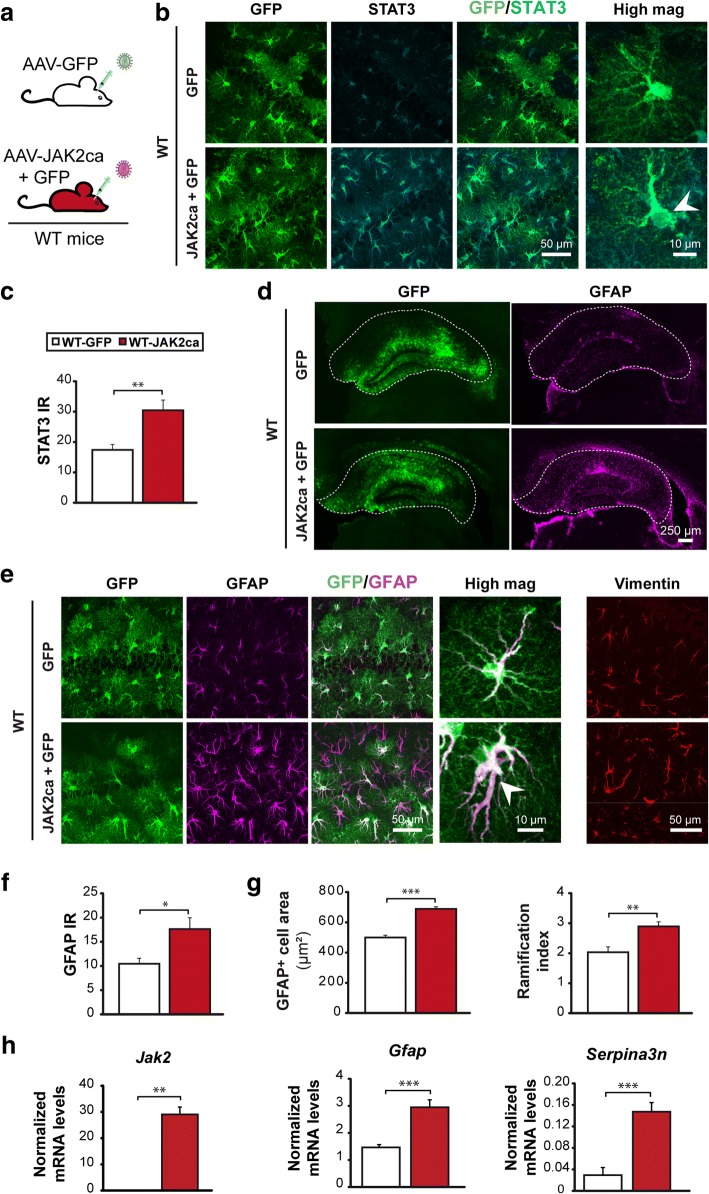
JAK2ca activates the JAK2-STAT3 pathway and induces astrocyte reactivity. **a**, WT mice were injected in the hippocampus with AAV-GFP alone (*N* = 6) or AAV-JAK2ca + AAV-GFP (JAK2ca + GFP, *N* = 6) at the same total viral titer and were studied 1–2 months later. **b**, Confocal images of hippocampal sections, stained for GFP (green) and STAT3 (cyan) in WT-GFP or WT-JAK2ca mice. JAK2ca induces STAT3 upregulation and nuclear accumulation in astrocytes, indicating STAT3 activation (arrowhead). **c**, STAT3 IR quantification in astrocyte nucleus from images in **b**. **d**, Representative low magnification images showing the transduced area (GFP^+^, green) and corresponding GFAP staining (magenta) in the hippocampus of WT-GFP and WT-JAK2ca mice. JAK2ca increases GFAP levels in a large part of the hippocampus (outlined). **e**, Confocal images of astrocytes stained for GFP (green), GFAP (magenta) and vimentin (red) in WT-GFP and WT-JAK2ca mice. JAK2ca increases GFAP and vimentin expression in hippocampal astrocytes and induces morphological changes. **f**, GFAP IR is increased by 70% in JAK2ca-injected hippocampus. **g**, Sholl analysis applied to GFAP-labelled astrocytes shows that reactive astrocytes in WT-JAK2ca mice have a larger domain area and a higher ramification index, a measure of cell complexity. **h**, RT-qPCR analysis was performed on acutely sorted hippocampal astrocytes from WT-GFP and WT-JAK2ca mice. *Jak2, Gfap* and *Serpina3n* are significantly overexpressed in WT-JAK2ca astrocytes. *N* = 7/group. **c**, **f**, **g**, Student *t* test. **h**, *Gfap, Serpina3n*: Student *t* test, *Jak2:* Mann-Whitney test. * *p* < 0.05, ** *p* < 0.01, *** *p* < 0.001

To this end, we first studied whether activation of the JAK2-STAT3 pathway by JAK2ca was sufficient to induce astrocyte reactivity in the hippocampus of WT mice (Fig. 7a). As expected, we found that JAK2ca significantly increased STAT3 immunoreactivity in hippocampal astrocytes (Fig. 7b, c). Importantly, immunoreactivity for GFAP was increased in a large part of the hippocampus of WT-JAK2ca mice (Fig. 7d-f). JAK2ca-astrocytes also overexpressed vimentin (Fig. 7e) and had enlarged soma with tortuous processes (Fig. 7e, g). RT-qPCR analysis on acutely isolated astrocytes from WT-JAK2ca or WT-GFP mice confirmed that JAK2ca significantly increased mRNA levels of reactive genes (*Gfap, serpina3n*) in astrocytes (Fig. 7h). Overall, activation of the JAK2-STAT3 pathway in astrocytes was sufficient to induce morphological and molecular hallmarks of reactivity in the complete absence of pathological environment.

**Fig. 8:**
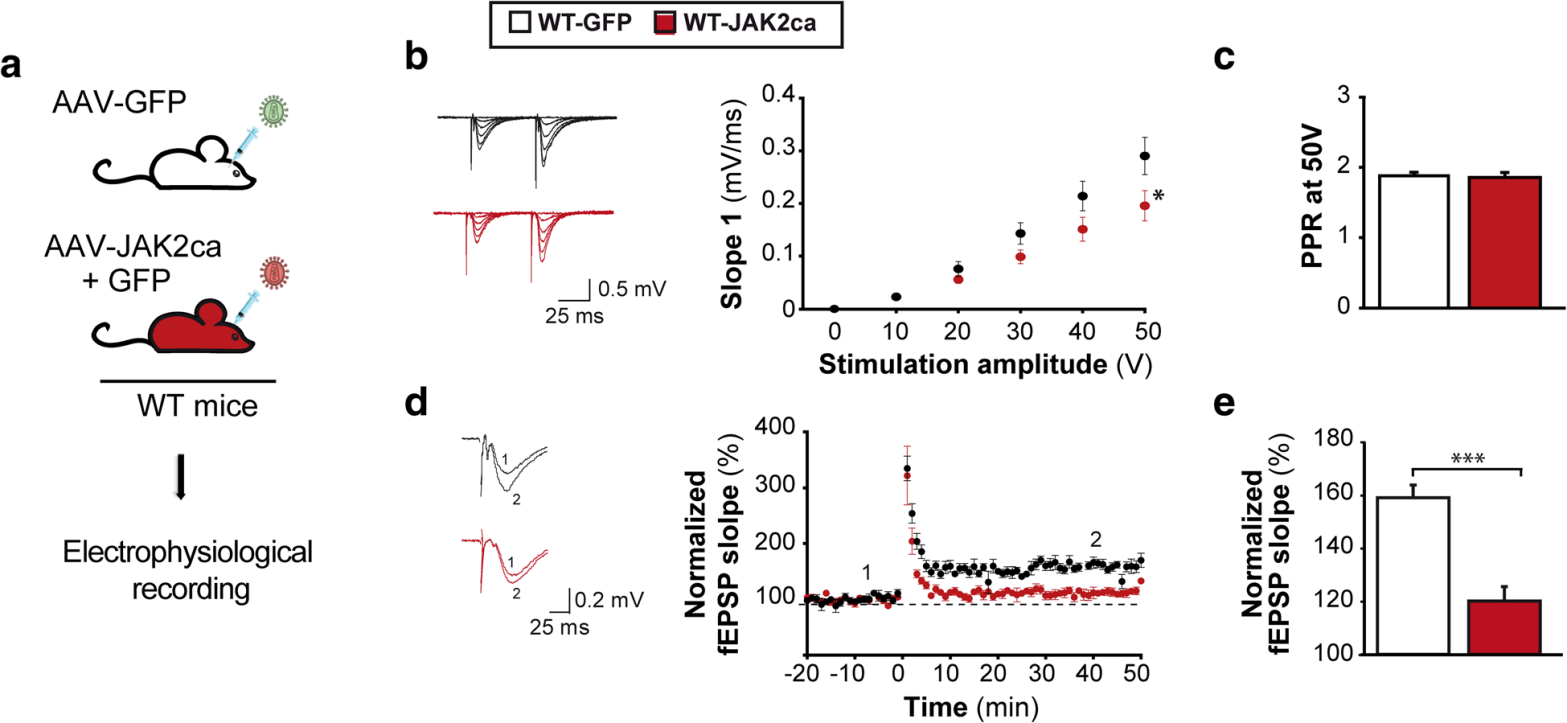
JAK2ca-induced astrocyte reactivity is sufficient to alter synaptic transmission and long-term plasticity in WT mice. **a**, Acute hippocampal slices were prepared from the hippocampus of 4–6 month-old WT-GFP and WT-JAK2ca mice. **b**-**c**, Representative paired-pulse stimulation traces for WT-GFP and WT-JAK2ca mice (100 ms interval). JAK2ca shifts the input/output relationship to the right, reducing the strength of basal glutamatergic transmission (**b**, Two way ANOVA and Bonferroni test) without impacting release probability, as revealed by unchanged PPR at 50 V (**c**, Student *t* test). *N* = 15–13. **d**, Representative (left) and average (right) fEPSPs before (1) and after (2) HFS protocol. LTP is impaired in WT-JAK2ca mice. **e**, Bar graph representing normalized fEPSP slopes 40 to 50 min post HFS. *N* = 8 in each group. Student *t* test. * *p* < 0.05, *** *p* < 0.001

We then recorded synaptic activity in the infected GFP^+^ hippocampal CA1 region of WT-GFP and WT-JAK2ca mice (Fig. 8a). JAK2ca-mediated astrocyte reactivity impaired basal glutamatergic synaptic transmission (Fig. 8b) but it did not impact the paired-pulse ratio (Fig. 8c). Strikingly, HFS protocol failed to induce LTP in JAK2ca mice, as in 3xTg mice, while it induced a 60% increase in fEPSP in WT-GFP mice (Fig. 8d, e), showing that JAK2ca causes significant deficits in long-term synaptic plasticity. Taken together, our results show that JAK2-STAT3-mediated astrocyte reactivity induces synaptic dysfunction in the hippocampus, and is a potent target for synaptic restoration in AD.

## Discussion

**Fig. 9:**
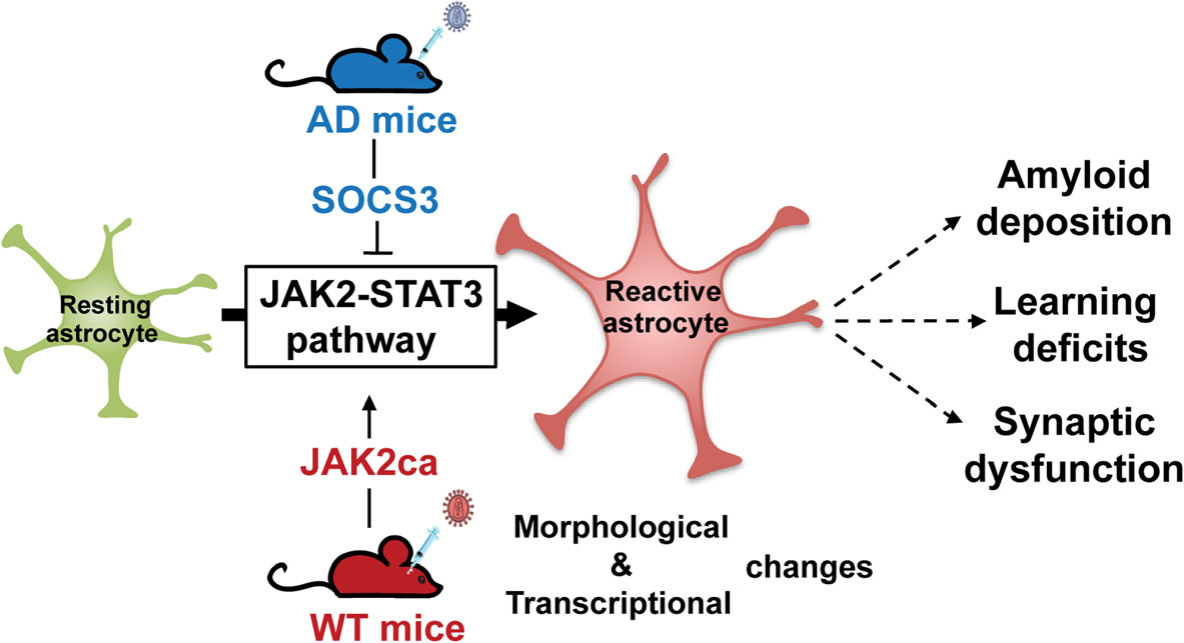
The JAK2-STAT3 pathway is a master regulator of astrocyte reactivity that contributes to AD deficits*.* SOCS3-mediated inhibition of this cascade in AD mouse models blocked and even reversed morphological and molecular hallmarks of reactivity. Conversely, activation of the JAK2-STAT3 pathway by viral gene transfer of JAK2ca in WT mice was sufficient to induce those hallmarks. Inhibition of this cascade in AD mice reduced amyloid deposition, deficits in spatial learning and synaptic dysfunction, showing that reactive astrocytes significantly contribute to AD pathological outcomes

In this study, we combined astrocyte-targeted viral gene transfer, histology, biochemical analysis, cell sorting, astrocyte-specific transcriptomics, behavioral analysis and electrophysiology, in mouse models of AD and naïve WT mice. We used two transgenic mouse models of AD that recapitulate amyloid deposition, synaptic and behavioral alterations characteristic of AD, as well as progressive astrocyte reactivity including morphological and molecular changes. We found that inhibition of the JAK2-STAT3 pathway normalizes several features of astrocyte reactivity and improves three key pathological hallmarks in AD mouse models, showing that reactive astrocytes have deleterious effects in AD (Fig. 9).

### The JAK2-STAT3 pathway controls astrocyte reactivity

We previously reported that STAT3 is activated in reactive astrocytes in several mouse and primate models of ND [8], and the active, phosphorylated form of STAT3 is found in some hippocampal GFAP^+^ astrocytes in AD patients [81]. STAT3 is also activated in reactive astrocytes in acute CNS diseases such as ischemia, or spinal cord injury [12, 61]. Many molecular triggers are able to activate STAT3 (e.g. cytokines, growth factors, as well as ephrins [80] or the Notch pathway [41]). Therefore, despite the variety of pathological stimuli associated with these disorders, they appear to converge, either directly or indirectly, on STAT3, making STAT3 activation a universal feature of astrocyte reactivity.

We used viral gene transfer in the mouse brain to inhibit or activate the JAK2-STAT3 pathway in astrocytes and identified this cascade as a master regulator of astrocyte reactivity. We show that overexpression of a constitutively active form of JAK2 in WT astrocytes is sufficient to trigger robust astrocyte reactivity in the hippocampus, in the absence of pathological stimulus. By expressing the inhibitor SOCS3 in astrocytes, we also showed that the JAK2-STAT3 pathway controls astrocyte reactivity in two mouse models of AD. Interestingly, SOCS3 was not only able to prevent, but also to reverse astrocyte reactivity in the hippocampus of aged APP mice. Thus, activation of the JAK2-STAT3 pathway is necessary both for the induction and persistence of many aspects of astrocyte reactivity (Fig. 9). Contrary to conditional approaches used in acute injury models [4, 26, 61], our SOCS3-based strategy to block reactivity has the advantage of inhibiting only the JAK-dependent effects of STAT3, without impacting other non-canonical functions of STAT3 that do not require phosphorylation on Tyrosine 705 [12]. In addition, our virus-based method provides a local and time-controlled inhibition of the JAK-STAT3 pathway, avoiding peripheral or developmental side effects. SOCS3 is a specific inhibitor of the JAK-STAT3 cascade thanks to its dual recognition of JAK and the phosphorylated motif on the cytokine receptor [6, 35]. We controlled that SOCS3 overexpression did not affect other signaling cascades. We did not find evidence for ERK or STAT1 activation in APP astrocytes and no further effect of SOCS3, suggesting that STAT3 is the main transcription factor regulated by SOCS3 in our conditions.

We found that SOCS3 regulates many genes linked to inflammation and immunity that are induced in astrocytes of AD mouse models (our data and [45, 62]) and in astrocytes of AD patient brains [62, 73]. In particular, several genes encoding cytokines and complement factors, which are associated with synaptic alterations and molecular defects characteristic of AD [25, 45, 78], were down-regulated by SOCS3. Interestingly, the induction of some SOCS3-regulated genes was shown to be dependent on STAT3, in a model of spinal cord injury in mice with an astrocyte-specific knock out of STAT3 [4]. Our study is of high therapeutic relevance for AD as it identifies the cascade controlling the transcription of neuroinflammatory genes by astrocytes. It will be important to measure how those inflammatory mediators are impacted by SOCS3 at the protein level.

Besides the JAK2-STAT3 pathway, other signaling cascades were reportedly activated during ND [7, 33]. But only a few cascades have been specifically manipulated in astrocytes to test their requirement for reactivity during AD. Constitutive activation of calcineurin in APP mice decreases astrocyte reactivity [19]. Surprisingly, the opposite manipulation of the calcineurin/Nuclear Factor of Activated T-cells (NFAT) pathway by expression of a blocking peptide in astrocytes also attenuates reactivity in the same mouse model [22], questioning the regulatory action of this pathway. Activation of the Nuclear Factor of κ light polypeptide gene enhancer in B-cells (NF-κB) pathway in astrocytes, through a conditional knock-out of the IκBα inhibitor or by expression of a constitutively active IKK, increases glial reactivity in the cortex and hippocampus of WT and AD mice [44, 60]. But, to our knowledge, the specific inhibition of this cascade in astrocytes has not been performed in AD models. Overall, modulation of calcineurin/NFAT or NF-κB pathways appear to have inconsistent, transient or moderate effects on astrocyte reactivity in AD models. On the contrary, we studied two mouse models of AD (APP and 3xTg) and observed consistent effects of JAK2-STAT3 pathway modulation on astrocyte reactivity. Importantly, these effects were remarkably stable over time, lasting up to 9 months post-injection.

The concept of reactive astrocyte heterogeneity is emerging [3, 47], especially with the recent description of A1 and A2 subtypes of reactive astrocytes [48, 68, 76, 84]. Reactive astrocytes may thus form discrete subtypes with specific molecular and functional properties. The precise signaling pathways controlling these reactive states are still unknown. Unexpectedly, we found that SOCS3 regulates the expression of pan, A1 and A2 specific genes. In fact, the core WGCNA module of SOCS3-regulated genes contained all types of reactive markers. These results suggest that SOCS3 mediates a global inhibition of astrocyte reactivity, which operates beyond specific classes of reactive astrocytes. In this study, we did not explore the effects of SOCS3 in WT astrocytes, it will be interesting to see whether SOCS3 also regulates the transcriptome of astrocytes and some of their functions when they are not in an AD pathological environment.

Surprisingly, we found that inhibition of astrocyte reactivity in APP mice by SOCS3 did not impact the molecular and functional features of microglial cells. Microglia and astrocytes are engaged in complex bidirectional communications [25]. In particular, reactive microglial cells are reported to play a key role in triggering astrocyte reactivity in inflammatory conditions [70]. Our results, suggest that, at least in AD, reactive astrocytes do not significantly regulate microglial activation. Microglial cells may be already strongly activated by toxic amyloid proteins and neuronal dysfunction as occurring in AD.

### Reactive astrocytes contribute to pathological outcomes in AD models

We found that inhibition of astrocyte reactivity by SOCS3 reduces the number of amyloid plaques in APP mice, a key pathological hallmark of AD. Intriguingly, the average size of plaques and the overall size distribution were not changed, suggesting that SOCS3 reduces amyloid seeding into plaques, but once amyloid plaques form, their growth and evolution are not impacted. Amyloid production, aggregation and clearance involve several brain cell types, including neurons that produce the bulk of Aβ peptides and microglial cells that actively degrade amyloid plaques. Astrocytes may produce low levels of Aβ as well [46] and are able to degrade amyloid plaques [37, 79, 82]. However, our FACS analysis evidenced no Aβ phagocytosis in astrocytes, and RNAseq data showed no increase in the expression of phagocytic receptors in astrocytes, suggesting that SOCS3-astrocytes do not contribute to Aβ phagocytosis. In addition, we ruled out microglial involvement in enhanced Aβ clearance in the SOCS3 group. Last, stable levels of soluble Aβ40 and Aβ42, as well as proteins involved in APP metabolism, suggest that Aβ production is not altered by JAK2-STAT3 pathway modulation in astrocytes. The molecular and cellular mechanisms responsible for the reduced number of amyloid plaques with SOCS3 are still unknown. Astrocytes were recently shown to contribute to Aβ elimination through the glymphatic system, which allows perivascular drainage of interstitial fluid to remove soluble brain waste [29]. SOCS3 could improve this clearance mechanism, thereby diverting soluble Aβ from aggregating into plaques. It is however quite difficult to test this hypothesis, as available methods to probe the glymphatic system lack spatial resolution to quantify SOCS3 local effects in the hippocampus.

We also report that SOCS3-mediated inhibition of astrocyte reactivity restores spatial learning on the Morris water maze, without correcting deficits in memory retrieval at 72 h. It is striking that expression of SOCS3 in only 25% of hippocampal astrocytes is able to restore spatial learning. Spatial learning is a hippocampal-dependent task [56], and it is now well documented that astrocytes are able to regulate hippocampal circuits and impact behavior [1, 66, 69]. However, the local action of AAV on hippocampal astrocytes may explain why long term memory was not corrected by AAV-SOCS3 in APP mice, because storage and retrieval of spatial memories mostly involve neocortical regions, which are not targeted by AAVs [52]. The finding that hippocampal astrocyte reactivity contributes to learning deficits in APP mice and is amenable to restoration by SOCS3 opens new therapeutic applications for AD.

Further demonstrating SOCS3 therapeutic potential, we found that its expression in astrocytes fully restored early synaptic and LTP alterations in 3xTg mice. Partial reduction in astrocyte reactivity by NFAT inhibition also improves synaptic transmission in aged APP mice [22]. Here, we uncover an implication of reactive astrocytes in the initial stages of synaptic dysfunction, before overt amyloid and Tau pathology, stressing the importance of astrocytes as therapeutic targets for AD. In accordance, we found that induction of astrocyte reactivity by JAK2ca was sufficient to induce synaptic deficits in WT mice, similar to those observed in 3xTg mice. Astrocytes regulate synaptic transmission by promoting ion homeostasis, recycling neurotransmitters or releasing active molecules such as gliotransmitters [5, 64, 65]. Some of these mechanisms are altered in AD mouse models [7, 15, 31] and could be restored by SOCS3. In addition, mRNA levels of several synaptotoxic complement factors [45, 78] were reduced by SOCS3. It is thus possible that SOCS3 normalizes several aspects of neuron-astrocyte interactions at the synapse, and further experiments will be needed to identify the exact mechanisms at play. Our model of JAK2ca-induced astrocyte reactivity will be helpful to isolate astrocyte-specific mechanisms of synaptic impairment.

The fact that JAK2-STAT3-mediated astrocyte reactivity contributes to three AD pathological outcomes contrasts with the beneficial effects of astrocytic STAT3 in spinal cord injury [4] or lack of effects after chemical lesions [58]. It is possible that some, but not all, astrocyte functions are regulated by this pathway. Depending on the disease context, STAT3-mediated astrocyte reactivity may thus have variable effects.

## Conclusion

We show that the JAK2-STAT3 pathway is a core signaling cascade for the induction and maintenance of astrocyte reactivity. This pathway controls key morphological features and coordinates gene expression in reactive astrocytes. The JAK2-STAT3 pathway is a potent molecular target to establish the overall role of reactive astrocytes in CNS diseases. Our results show that in AD, reactive astrocytes are mostly deleterious, contributing to amyloid deposition, spatial learning deficits and synaptic dysfunction. Blocking astrocyte reactivity via the JAK2-STAT3 pathway offers new therapeutic opportunities for AD.

## Additional files

Additional file 1: **Figure S1.** AAV infect astrocytes selectively. To validate astrocyte tropism of the AAVs used in our study, an AAV2/9 encoding GFP was injected in the hippocampus of WT mice. GFP^+^ cells co-express the astrocytic marker GFAP and S100β, but not NeuN, IBA1, Olig2 and MBP, which are markers of neurons, microglial cells, cells of the oligodendrocyte lineage and myelinating oligodendrocytes, respectively. Astrocyte tropism of these vectors was confirmed in AD mice as well (See colocalization of GFP with GFAP in Figs. 1b and S2). (TIF 14477 kb)

Additional file 2: **Table S1.** Sequences of primers used for qPCR. (DOCX 17 kb)

Additional file 3: **FigureS2.** Unlike STAT3, STAT1 and Erk are not activated in APP astrocytes. Confocal images of stained hippocampal sections from 12-month-old WT-GFP, APP-GFP and APP-SOCS3 mice. **a**, GFP^+^ astrocytes (green) stained for GFAP (magenta) and STAT3 (cyan). APP astrocytes are reactive (hypertrophic and GFAP overexpression). They display STAT3 nuclear accumulation. SOCS3 reduces GFAP and STAT3 expression in APP mice, even around amyloid plaques (star). **b**, Quantification of STAT3 immunoreactivity in astrocyte soma. *N* = 5–7/group. One way ANOVA and Tukey’s post hoc test. *** *p* < 0.001. **c-d**, Sections stained in magenta for STAT1 (**c**) or P-ERK (**d**), DAPI (blue) and GFP (green). STAT1 and P-ERK are not induced in APP reactive astrocytes while CNTF induces significant STAT1 nuclear accumulation (**c**) and LPS triggers ERK phosphorylation (**d**). DAPI stains nuclei as well as amyloid plaques. Representative images from *N* = 4–6/group. (TIF 15218 kb)

Additional file 4: **Figure S3.** Validation of astrocyte sorting. Normalized expression of cell type specific genes. GFP^+^ astrocytes are enriched in astrocyte markers while GFP^−^ cells, which comprise uninfected astrocytes, neurons, microglial cells and oligodendrocyte precursor cells (OPC) are enriched in other cell type markers. Oligodendocyte markers are undetectable due to the myelin removal step. *N* = 3–7/group. Wald test. * *p* < 0.05, ** *p* < 0.01, *** *p* < 0.001, ^#^ *p* < 10^− 20^, ^###^ *p* < 10^− 40^. (TIF 12358 kb)

Additional file 5: **Table S2.** WGCNA: Top 20 most connected genes regulated by SOCS3 in APP astrocytes. Three of the top 20 hub genes, highlighted in bold, are pan or A1 reactive astrocyte genes. (DOCX 17 kb)

Additional file 6: **Figure S4.** SOCS3 regulates gene networks linked to reactivity in APP astrocytes. The top 100 most connected genes of the WGCNA module were analyzed for protein-protein interaction networks with STRING. Groups of proteins related to complement system and inflammation, cytoskeleton and cell adhesion were found co-regulated by SOCS3 (circles). Protein-protein interaction *p* value < 10^− 6^. (TIF 54618 kb)

Additional file 7: **Figure S5.** Modulation of the JAK2-STAT3 pathway does not change expression of proteins involved in Aβ production and clearance in APP mice. Representative western blottings on protein homogenates prepared from WT-GFP, APP-GFP, APP-SOCS3 and APP-JAK2ca mice. The expression of human (hAPP) (**a**), BACE1 (**b**), IDE (**c**) and ApoE (**d**) are stable across the groups (protein levels are normalized by actin or tubulin α). *N* = 3–8/group. **a**, Student t test, **b-d**, ANOVA or Kruskall-Wallis tests. (TIF 10130 kb)

Additional file 8: **Table S3.** Expression of phagocytic receptors and genes in astrocytes RNAseq analysis of astrocytes shows that most genes involved in Aβ phagocytosis are expressed at similar levels in all groups. Only mRNA levels for three Fc receptors are down-regulated by SOCS3. Data are presented as normalized mRNA levels +/-SEM. ^##^ *p* < 0.01 versus WT-GFP, ** *p* < 0.01, *** *p* < 0.001 versus APP-GFP. Wald test. (DOCX 18 kb)
