# Supplementary figures and images for: Modulation of astrocyte reactivity improves functional deficits in mouse models of Alzheimer’s disease
Source: Acta Neuropathol Commun. 2018 Oct 16;6:104. doi: 10.1186/s40478-018-0606-1 (PMC6190663; doi:10.1186/s40478-018-0606-1)

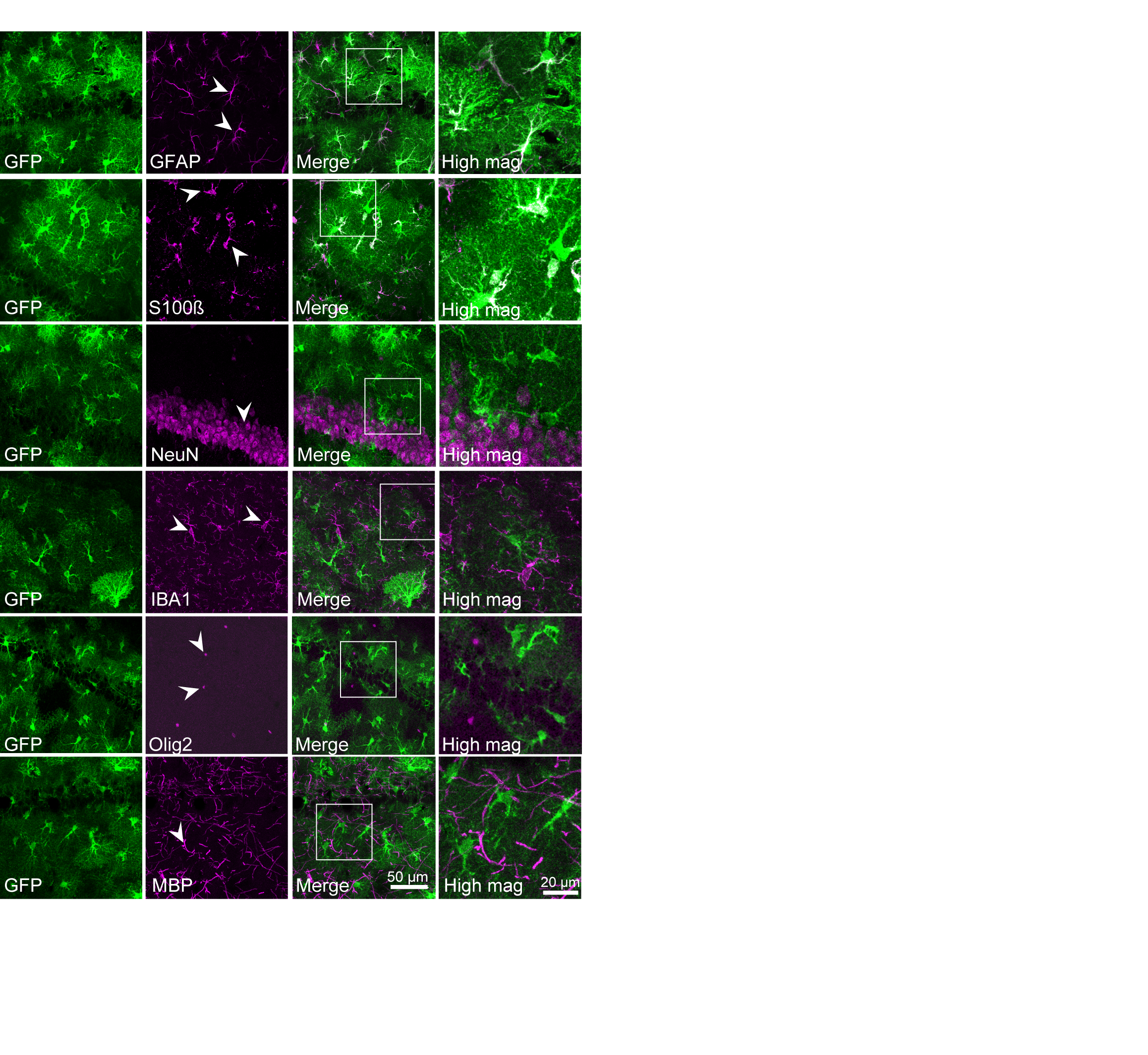

Supplement: Supplementary file 1 — Figure S1. AAV infect astrocytes selectively. To validate astrocyte tropism of the AAVs used in our study, an AAV2/9 encoding GFP was injected in the hippocampus of WT mice. GFP+ cells co-express the astrocytic marker GFAP and S100β, but not NeuN, IBA1, Olig2 and MBP, which are markers of neurons, microglial cells, cells of the oligodendrocyte lineage and myelinating oligodendrocytes, respectively. Astrocyte tropism of these vectors was confirmed in AD mice as well (See colocalization of GFP with GFAP in Figs. 1b and S2). (TIF 14477 kb) [file 40478_2018_606_MOESM1_ESM.tif]

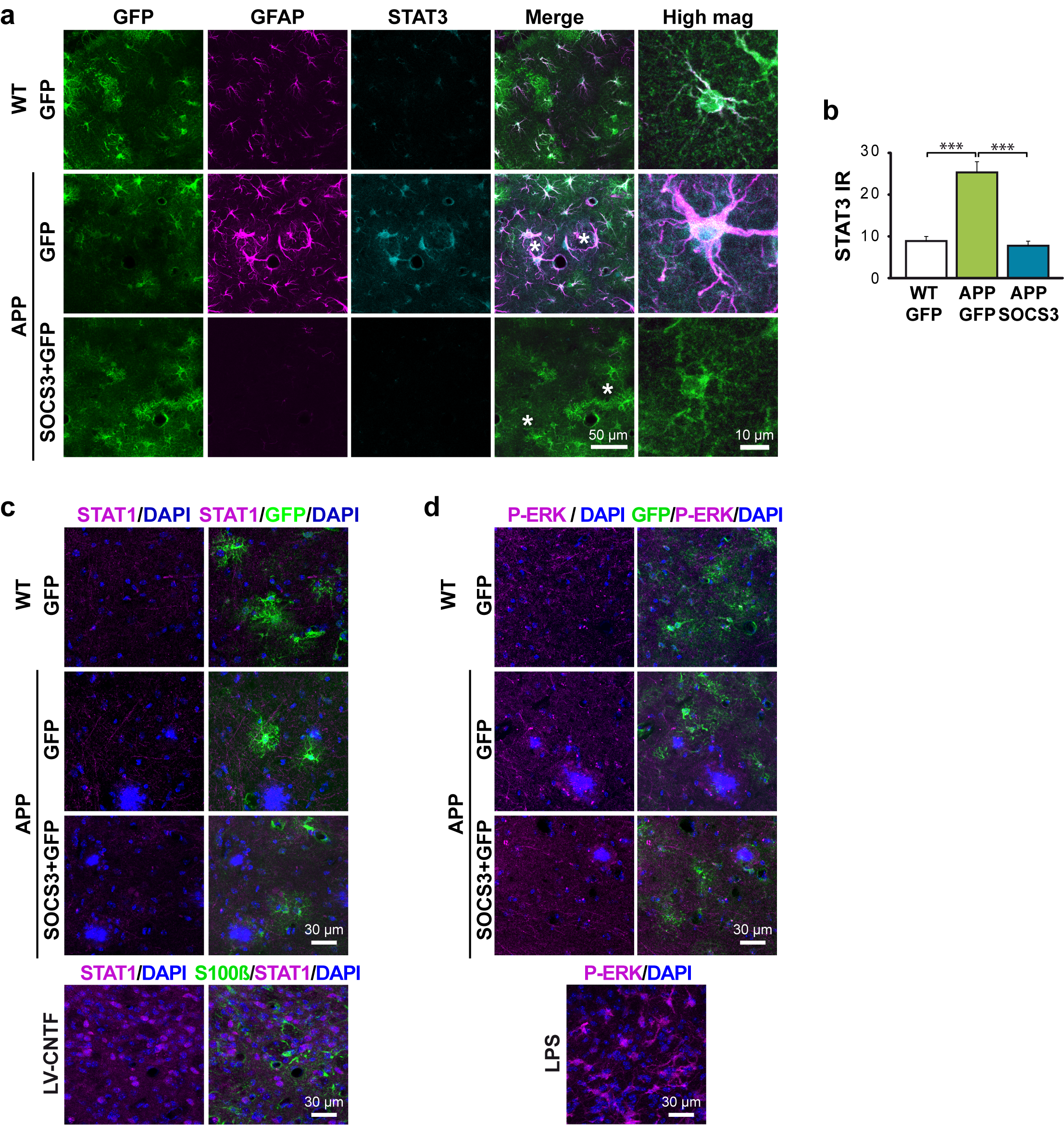

Supplement: Supplementary file 3 — FigureS2. Unlike STAT3, STAT1 and Erk are not activated in APP astrocytes. Confocal images of stained hippocampal sections from 12-month-old WT-GFP, APP-GFP and APP-SOCS3 mice. a, GFP+ astrocytes (green) stained for GFAP (magenta) and STAT3 (cyan). APP astrocytes are reactive (hypertrophic and GFAP overexpression). They display STAT3 nuclear accumulation. SOCS3 reduces GFAP and STAT3 expression in APP mice, even around amyloid plaques (star). b, Quantification of STAT3 immunoreactivity in astrocyte soma. N = 5–7/group. One way ANOVA and Tukey’s post hoc test. *** p < 0.001. c-d, Sections stained in magenta for STAT1 (c) or P-ERK (d), DAPI (blue) and GFP (green). STAT1 and P-ERK are not induced in APP reactive astrocytes while CNTF induces significant STAT1 nuclear accumulation (c) and LPS triggers ERK phosphorylation (d). DAPI stains nuclei as well as amyloid plaques. Representative images from N = 4–6/group. (TIF 15218 kb) [file 40478_2018_606_MOESM3_ESM.tif]

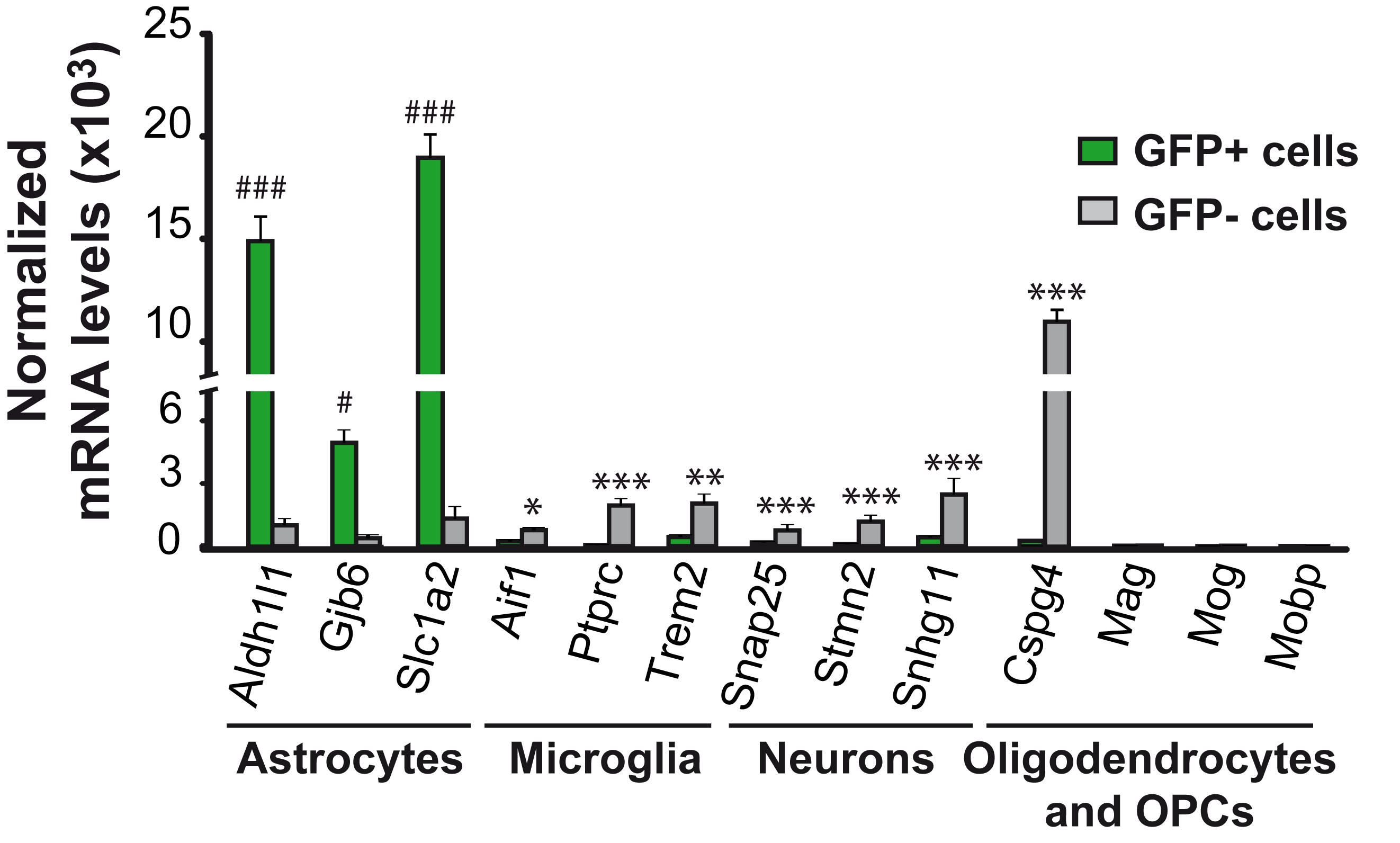

Supplement: Supplementary file 4 — Figure S3. Validation of astrocyte sorting. Normalized expression of cell type specific genes. GFP+ astrocytes are enriched in astrocyte markers while GFP− cells, which comprise uninfected astrocytes, neurons, microglial cells and oligodendrocyte precursor cells (OPC) are enriched in other cell type markers. Oligodendocyte markers are undetectable due to the myelin removal step. N = 3–7/group. Wald test. * p < 0.05, ** p < 0.01, *** p < 0.001, # p < 10− 20, ### p < 10− 40. (TIF 12358 kb) [file 40478_2018_606_MOESM4_ESM.tif]

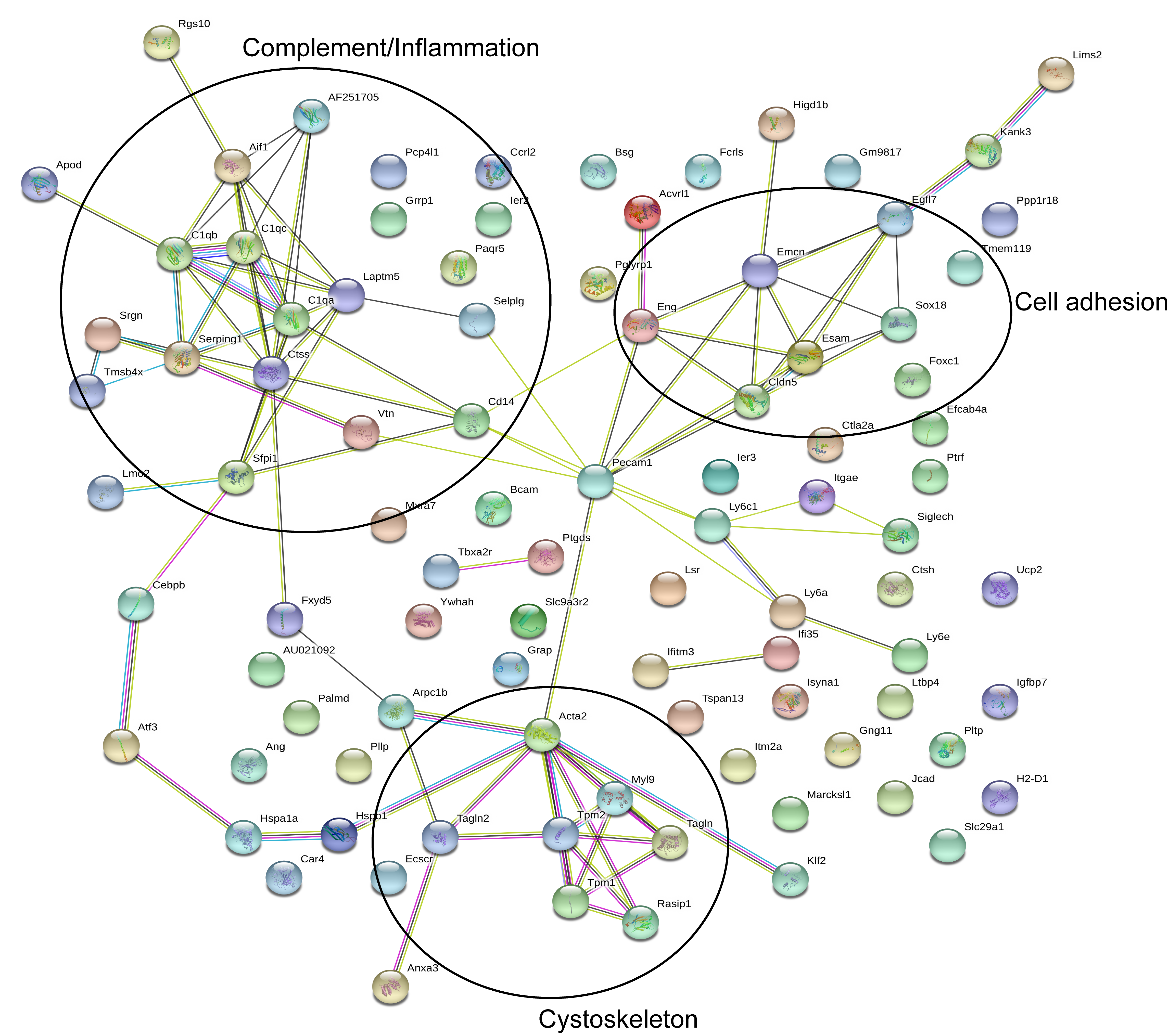

Supplement: Supplementary file 6 — Figure S4. SOCS3 regulates gene networks linked to reactivity in APP astrocytes. The top 100 most connected genes of the WGCNA module were analyzed for protein-protein interaction networks with STRING. Groups of proteins related to complement system and inflammation, cytoskeleton and cell adhesion were found co-regulated by SOCS3 (circles). Protein-protein interaction p value < 10− 6. (TIF 54618 kb) [file 40478_2018_606_MOESM6_ESM.tif]

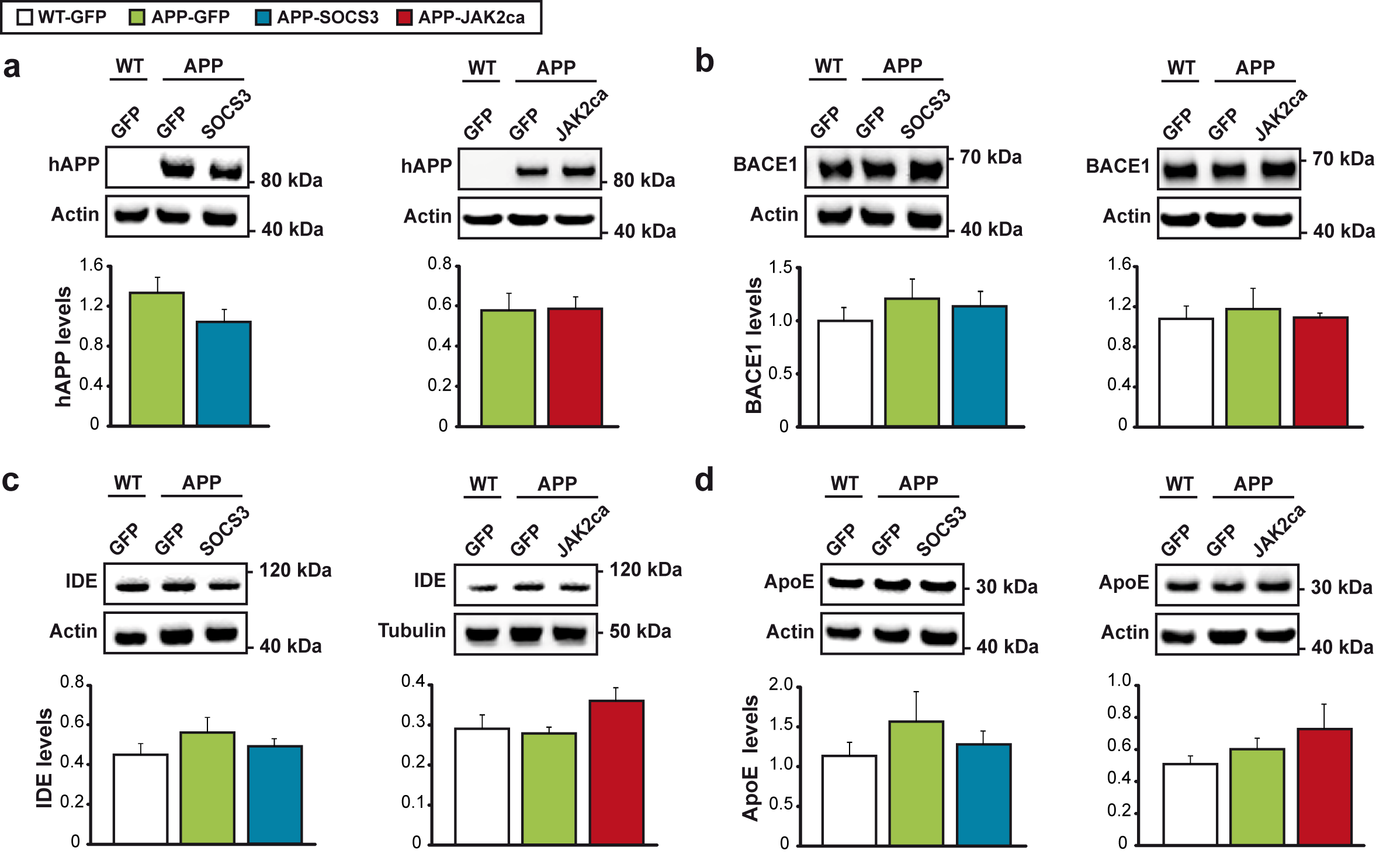

Supplement: Supplementary file 7 — Figure S5. Modulation of the JAK2-STAT3 pathway does not change expression of proteins involved in Aβ production and clearance in APP mice. Representative western blottings on protein homogenates prepared from WT-GFP, APP-GFP, APP-SOCS3 and APP-JAK2ca mice. The expression of human (hAPP) (a), BACE1 (b), IDE (c) and ApoE (d) are stable across the groups (protein levels are normalized by actin or tubulin α). N = 3–8/group. a, Student t test, b-d, ANOVA or Kruskall-Wallis tests. (TIF 10130 kb) [file 40478_2018_606_MOESM7_ESM.tif]
