# Supplementary material for: Modulation of astrocyte reactivity improves functional deficits in mouse models of Alzheimer’s disease
Source: Acta Neuropathol Commun. 2018 Oct 16;6:104. doi: 10.1186/s40478-018-0606-1 (PMC6190663; doi:10.1186/s40478-018-0606-1)
Supplement: Supplementary file 2 — Table S1. Sequences of primers used for qPCR. (DOCX 17 kb) [file 40478_2018_606_MOESM2_ESM.docx]

| **Gene** | **Forward primer** | **Reverse primer** |
| --- | --- | --- |
| ***Actin*** | AGAGGGAAATCGTGCGTGAC | CGATAGTGATGACCTGACCGT |
| ***ApoE*** | CTGAACCGCTTCTGGGATTACCTG | CATAGTGTCCTCCATCAGTGCCGTC |
| ***C1qb*** | CCACGCAACGGCAAGTTCAC | CGGCCACGAACGAGATTCAC |
| ***Ctss*** | GGCATGAACGATATGGGAG | TCAGGCAATGTCCGATTAGA |
| ***Eef1a1*** | CTACCCTCCACTTGGTCGCTT | GCAACTGTCTGCCTCATGTCAC |
| ***Gfap*** | ACGACTATCGCCGCCAACT | GCCGCTCTAGGGACTCGTTC |
| ***Jak2*** | GTCTTGGGATGGCGGTGTTAG | ATTGCTGAATGAATCTGCGAAATCT |
| ***Rpl13a*** | CTGAAGCCTACCAGAAAGTTTGC | GGTACTTCCACCCGACCTCAT |
| ***Serpina3n*** | CAACCTTACAGGCCAACCCAT | GGGCACCAAGTAGTCCTAGATGCT |
| ***Tmem119*** | GTGTCTAACAGGCCCCAGAA | AGCCACGTGGTATCAAGGAG |
| ***Trem2*** | AATGGGAGCACAGTCATCGCAGA | ACTGGTAGAGGCCCGCGTCAC |

#### **Table S1. Sequences of primers used for qPCR.**
