# Supplementary material for: Modulation of astrocyte reactivity improves functional deficits in mouse models of Alzheimer’s disease
Source: Acta Neuropathol Commun. 2018 Oct 16;6:104. doi: 10.1186/s40478-018-0606-1 (PMC6190663; doi:10.1186/s40478-018-0606-1)
Supplement: Supplementary file 5 — Table S2. WGCNA: Top 20 most connected genes regulated by SOCS3 in APP astrocytes. Three of the top 20 hub genes, highlighted in bold, are pan or A1 reactive astrocyte genes. (DOCX 17 kb) [file 40478_2018_606_MOESM5_ESM.docx]

| **Symbol** | **Gene name** |
| --- | --- |
| *Fxyd5 // Mir7050* | FXYD domain-containing ion transport regulator 5 // microRNA 7050 |
| *Pcp4l1* | Purkinje cell protein 4-like 1 |
| *Acvrl1* | activin A receptor, type II-like 1 |
| *Igfbp7* | insulin-like growth factor binding protein 7 |
| *Ly6e* | lymphocyte antigen 6 complex, locus E |
| *Itm2a* | integral membrane protein 2A |
| *Gm9817* | predicted gene 9817 |
| *Arpc1b* | actin related protein 2/3 complex, subunit 1B |
| ***Serping1*** | **serine (or cysteine) peptidase inhibitor, clade G, member 1** |
| *Ctsh* | cathepsin H |
| *Apod* | apolipoprotein D |
| *Slc9a3r2* | solute carrier family 9 (sodium/hydrogen exchanger), member 3 regulator 2 |
| *Lmo2* | LIM domain only 2 |
| *Tpm1* | tropomyosin 1, alpha |
| *Tmem119* | transmembrane protein 119 |
| *Rasip1 // Izumo1* | Ras interacting protein 1 // izumo sperm-egg fusion 1 |
| *Bsg* | basigin |
| ***Srgn*** | **serglycin** |
| *Ly6c1* | lymphocyte antigen 6 complex, locus C1 |
| ***Hspb1*** | **heat shock protein 1** |

#### **Table S2. WGCNA: Top 20 most connected genes regulated by SOCS3 in APP astrocytes.**

Three of the top 20 hub genes, highlighted in bold, are pan or A1 reactive astrocyte genes.
