# Supplementary material for: Modulation of astrocyte reactivity improves functional deficits in mouse models of Alzheimer’s disease
Source: Acta Neuropathol Commun. 2018 Oct 16;6:104. doi: 10.1186/s40478-018-0606-1 (PMC6190663; doi:10.1186/s40478-018-0606-1)
Supplement: Supplementary file 8 — Table S3. Expression of phagocytic receptors and genes in astrocytes RNAseq analysis of astrocytes shows that most genes involved in Aβ phagocytosis are expressed at similar levels in all groups. Only mRNA levels for three Fc receptors are down-regulated by SOCS3. Data are presented as normalized mRNA levels +/-SEM. ## p < 0.01 versus WT-GFP, ** p < 0.01, *** p < 0.001 versus APP-GFP. Wald test. (DOCX 18 kb) [file 40478_2018_606_MOESM8_ESM.docx]

| **Gene symbol** | **WT-GFP** | **APP-GFP** | **APP-SOCS3** |
| --- | --- | --- | --- |
| *Abca1* | 2727.5 ± 573.8 | 2316.3 ± 221.8 | 3022.1 ± 425.1 |
| *Apoe* | 1064461.8 ± 144708.2 | 1106705.9 ± 131395.1 | 1074356.3 ± 91250.2 |
| *Axl* | 2825.4 ± 391.5 | 3569.8 ± 687.3 | 3100.3 ± 433.1 |
| *Fcer1g* | 413.8 ± 76.4 | 1101.8 ± 422.0^##^ | 181.2 ± 37.2*** |
| *Fcgr2b* | 108.8 ± 52.4 | 232.2 ± 120.1 | 39.9 ± 5.0** |
| *Fcgr3* | 484.4 ± 100.4 | 1083.0 ± 386.2 | 194.9 ± 43.0*** |
| *Gulp1* | 273.1 ± 67.1 | 183.4 ± 40.7 | 247.1 ± 26.0 |
| *Itgav* | 1368.8 ± 374.8 | 907.2 ± 351.8 | 1029.6 ± 506.2 |
| *Itgb5* | 12739.4 ± 713.3 | 13516.9 ± 1493.5 | 12153.2 ± 722.4 |
| *Ldlr* | 1192.2 ± 374.4 | 1032.3 ± 431.3 | 1505.7 ± 423.3 |
| *Lrp1* | 2727.5 ± 299.6 | 2785.7 ± 230.1 | 2928.9 ± 302.3 |
| *Megf10* | 577.1 ± 171.6 | 361.3 ± 116.1 | 635.7 ± 173.3 |
| *Mertk* | 2358.5 ± 517.9 | 1987.6 ± 415.6 | 2077.9 ± 448.0 |

Table S3 Expression of phagocytic receptors and genes in astrocytes

RNAseq analysis of astrocytes shows that most genes involved in Aβ phagocytosis are expressed at similar levels in all groups. Only mRNA levels for three Fc receptors are down-regulated by SOCS3. Data are presented as normalized mRNA levels +/-SEM. ^##^ *p* < 0.01 versus WT-GFP, ****** *p* < 0.01, ******* *p* < 0.001 versus APP-GFP. Wald test.
